# Engineered Cu_3_P–ZnWO_4_ heterojunction integrated with porous polymer monolithic template for enhanced photocatalytic degradation of organic pollutants

**DOI:** 10.1038/s41598-025-29880-9

**Published:** 2025-12-01

**Authors:** Dhivya J., Lingesh Gopalakrishnan, Prabhakaran Deivasigamani, Akhila Maheswari Mohan

**Affiliations:** Department of Chemistry, School of Advanced Sciences, Vellore Institute of Technology (VIT), Vellore, Tamil Nadu 632014 India

**Keywords:** Metal oxides, Z-scheme heterojunction, Polymer template, Moxifloxacin drug, Visible light, Heterogeneous photocatalysis, Chemistry, Environmental sciences, Materials science, Nanoscience and technology

## Abstract

The strategic fabrication of efficient, renewable, and sustainable visible photon-responsive advanced heterogeneous photocatalysts is currently relevant for decontaminating pharmaceutical pollutants. Here, we report the fabrication of a unique Cu_3_P–ZnWO_4_ (CZ) heterojunction nanocomposite (NC) uniformly decorated onto a porous poly(EGDMA) monolith (PEM) template, which features a remarkable surface area, excellent structural integrity, and high porosity. Varying ratios of Cu_3_P to that of ZnWO_4_ reveal a sequence of Z-scheme heterostructured NCs, i.e., CZ-5, CZ-10, CZ-15, CZ-20, and CZ-25. The structurally engineered translucent PEM template and the CZ NCs decorated PEM are characterized by p-XRD, FT-IR, FE-SEM-EDAX, HR-TEM-SAED, VB-XPS, BET/BJH, UV–Vis-DRS, and PL analysis to confirm the formation of the desired photocatalyst with impressive structural and surface morphological features. The photocatalytic degradation efficiency shows that the CZ-20 NC-dispersed PEM (CZ-20@PEM) photocatalyst proffers robust photocatalytic performance for decontaminating moxifloxacin residues. Moreover, to determine the optimal conditions for fast and efficient photocatalysis, the influence of various analytical parameters, including solution pH (2–9), photocatalyst dosage (10–150 mg), pollutant concentration (10–50 ppm), oxidizers (KBrO_3_ & H_2_O_2_), and light intensities (150–300 W/m^2^) has been comprehensively studied. The CZ-20@PEM photocatalyst exhibits ≥ 99.4% moxifloxacin dissipation in ≤ 20 min, using 240 W/m^2^ visible light intensity. Based on VB-XPS analysis and trapping experiments, a feasible photocatalytic mechanism was proposed to clarify the reactive species predominantly participating in the photocatalytic process. This work demonstrates an efficient and sustainable approach for removing moxifloxacin drug residues, underscoring the potential of nanocomposite-encapsulated polymer monoliths as a next-generation photocatalytic platform for future water treatment applications.

**Supplementary Information:**

The online version contains supplementary material available at 10.1038/s41598-025-29880-9.

## Introduction

Environmental concerns are intensifying due to the continuous presence of pharmaceutical contaminants in water bodies, which adversely affect natural ecosystems and human populations alike. Consequential pharmaceutical residues, encompassing active pharmaceutical ingredients and persistent compounds, persist in the environment, contributing to pollution^1–3^. Moreover, the overutilization of pharmaceutical compounds poses a serious health hazard during effluent discharge through groundwater infiltration or surface runoff, threatening aquatic organisms and ecosystems and exacerbating antimicrobial resistance^4,5^. Fluoroquinolones, a category of broad-spectrum antimicrobials, find widespread utility for treating respiratory tract infections, urinary tract infections, seizures, intra-abdominal infections, etc. In this line, moxifloxacin is an advanced fluoroquinolone-based antimicrobial drug with excellent efficacy over anaerobic and gram-positive bacteria^6,7^. Moxifloxacin (MOX), a widely prescribed fluoroquinolone antibiotic, has raised concerns regarding its potential ecological risks at environmentally relevant concentrations, as evidenced by its frequent detection in aquatic environments. Reported levels of MOX in municipal wastewater range from 0.18 to 224 μg/L. Recent in silico studies further suggest that fluoroquinolones, including moxifloxacin, may inhibit SARS-CoV-2 replication due to their strong binding affinity for the virus’s main protease. However, conventional biological treatment processes exhibit significantly lower efficiency in removing MOX compared to other fluoroquinolones. MOX retains global recognition for its broad-spectrum antibacterial activity, earning inclusion in the World Health Organization’s list of essential medications, despite ongoing concerns. However, despite its extensive efficacy and therapeutic benefits, it faces considerable challenges stemming from improper disposal and unregulated use, which lead to bioaccumulation and pose health risks, including inhibition of neurotransmission, disruptions of the microbiota, peripheral neuropathy, ocular complications, musculoskeletal disorders, and reproductive dysfunctions^8,9^.

Heterogeneous photocatalytic processes are favoured to eradicate various persistent organic pollutants from aquatic environments due to the ease of photocatalyst separation and recyclability, which are crucial for large-scale industrial applications^10,11^. Heterogeneous photocatalysis-based advanced oxidation processes (AOPs) using metal oxides, such as TiO_2_, ZnO, ZnS, Fe_2_O_3_, WO_3_, and Bi_2_O_3_, have a long history in the literature^12^. However, employing metal oxides in photocatalysis has limitations, as they harness solar energy only in the ultraviolet (UV) spectrum, thereby preventing the utilization of the full spectrum of solar radiation, including vital visible light^13,14^. Furthermore, the rapid recombination of electron–hole (e^−^/h^+^) pairs generated during photocatalysis detrimentally affects overall process efficiency, hindering the breakdown of contaminants into harmless byproducts. Hence, to address this concern, several metal tungstates, such as Bi_2_WO_6_ and WO_3_, have been proposed due to their narrower band gaps, which enable increased visible-light absorption, improved charge separation, reduced electron–hole (e^−^/h^+^) recombination rates, and high photocatalytic activity, as well as versatility. In this context, ZnWO_4_, a wide-bandgap material, finds utility in various applications, including light-emitting diodes, water splitting, optical hole-burning lattice materials, sensors, and photocatalysis^15–17^.

Although ZnWO_4_ has demonstrated promising photocatalytic properties in various applications, it has certain limitations for pharmaceutical degradation due to its limited efficiency in utilizing visible light energy, attributed to its relatively wide bandgap that confines its absorption to ultraviolet (UV) light, in addition to a high e^−^/h^+^ recombination rate that jeopardizes the degradation process^18,19^. However, the photocatalytic performance of ZnWO_4_ can be amplified through doping with metal oxides, phosphides, and sulfides^20^. In this regard, metal phosphide nanomaterials, such as copper phosphide (Cu_3_P), can serve as effective dopants due to their extensive applications in energy storage, catalysis, sensors, optoelectronics, and biomedical devices^21^. Cu_3_P offers advantages stemming from its relative abundance, ensuring cost-effectiveness and environmental sustainability. Its remarkable ability to capture visible light enhances its light absorption, facilitating more active utilization of solar energy. Despite the numerous advantages of nanomaterials, potential toxicity and reusability issues arising from inefficient recovery pose a formidable challenge. Numerous Cu_3_P-/ZnWO_4_-based photocatalysts have been developed for environmental and energy-related applications. Copper phosphide (Cu_3_P) has been combined with various semiconductors to construct heterojunction photocatalysts, while zinc tungstate (ZnWO_4_) has been widely incorporated into hybrid systems to enhance photocatalytic degradation efficiency. For instance, including Cu_3_P/ZnIn_2_S_4_^22^, Cu_3_P/ZnSe^23^, ZnWO_4_/FeWO_4_^24^, and ZnWO_4_/CoWO_4_/g-C_3_N_4_^25^, have exhibited improved charge separation and visible-light-driven activity. Nevertheless, despite these advances, the development of a Cu_3_P/ZnWO_4_ heterojunction remains unexplored.

In this study, a novel Cu_3_P/ZnWO_4_ heterostructure was rationally designed as a visible light-responsive photocatalyst with enhanced photocatalytic efficiency. Additionally, recent studies on transition-metal phosphide and zinc tungstate cocatalysts were cited to provide methodological context, as summarized in the *Electronic Supplementary Material* (Table S1). To address reusability/recovery issues, we have investigated the immobilization of Cu_3_P–ZnWO_4_ NCs onto a porous polymer monolith with unique physico-chemical properties. The proposed new-age renewable photocatalyst offers easy fabrication, cost-effectiveness, high surface area, high porosity, and compatibility, thus making it an exceptional template for photocatalytic applications. Motivated by these unparalleled characteristics, we incorporated our synthesized Cu_3_P/ZnWO_4_ NCs onto a porous poly(EGDMA) monolith. To the best of our knowledge, the current work reports the first use of a Cu_3_P/ZnWO_4_ NC heterojunction uniformly dispersed within a structurally engineered porous polymer monolith template as a visible-light-responsive photocatalyst. The distinctive incorporation of the Cu_3_P/ZnWO_4_ heterostructure into the monolithic polymer framework enables rapid degradation of non-biodegradable, persistent antimicrobial drugs, including, but not limited to, moxifloxacin. The current work highlights the feasibility of tuning photocatalytic activity by varying the chemical and physical properties of the template material and fine-tuning the stoichiometric combination of Cu_3_P and ZnWO_4_. The work aims to acquire fresh mechanistic insights into the photocatalytic mechanism involved in the degradation of moxifloxacin and the degradation pathway leading to the formation of photoproducts.

## Experimental sections

### Chemicals and materials characterization

The comprehensive details regarding the chemicals, reagents, and solvents employed for photocatalyst synthesis and photocatalytic studies, as well as the instrumentation utilized for characterizing CZ NCs, PEM, and the CZ-20@photocatalyst, are provided in the *Electronic Supplementary Material*.

### Synthesis of Cu_3_P and ZnWO_4_

A simple co-precipitation technique was employed to synthesize copper phosphide (Cu_3_P), for which Cu(NO_3_)_2_·3H_2_O (0.1 M, 50 mL) was added dropwise to a NaOH solution (0.25 M, 100 mL) at constant stirring. A light blue precipitate of Cu(OH)_2_ formed immediately upon the addition of NaOH. After stirring the reaction mixture continuously for 2 h, the resulting precipitate was filtered, thoroughly washed with deionized water and ethanol to remove impurities, and subsequently dried in an oven at 70 °C. The dried Cu(OH)_2_ (0.5 g) was homogenized with 2 g of NaH_2_PO_4_ as a phosphorus source in a mortar and pestle, then calcined at 300 °C for 2 h with a ramp rate of 2 °C/min. After the calcination process, the greyish-black product was extensively rinsed with deionized water and ethanol to eliminate residual impurities, followed by Vacuum drying at 60 °C overnight to obtain purified Cu_3_P.

A hydrothermal methodology was employed to synthesize ZnWO_4_, for which Na_2_WO_4_ (0.5 M, 40 mL) was added dropwise to ZnCl_2_ (0.5 M, 40 mL) under continuous stirring for 2 h to ensure homogeneous mixing. The prepared solution was transferred to a 100 mL Teflon-lined autoclave and heated to 180 °C for 24 h. Once cooled naturally to room temperature, the resulting white solid was isolated via centrifugation and purified by successive washes with ethanol and deionized water. The product was then dried at 70 °C and further annealed at 500 °C for four hours to obtain crystalline ZnWO_4_ with enhanced thermal stability.

### Synthesis of Cu_3_P–ZnWO_4_ nanocomposites

For the synthesis of Cu_3_P–ZnWO_4_ nanocomposites (CZ NCs), various ratios of Cu_3_P and ZnWO_4_, i.e., 5:95, 10:90, 15:85, 20:80, 25:75, designated as CZ-5, CZ-10, CZ-15, CZ-20, and CZ-25, were mixed in 15 mL of ethanol and continuously stirred for 24 h, followed by ultrasonic treatment for 10 min. The resulting CZ NCs were oven-dried at 80 °C for 24 h to achieve complete dryness and stability. Finally, the NCs were finely ground to ensure uniformity and homogeneity of the obtained CZ NCs. The optimal CZ NC composition for visible-light-driven photocatalytic applications was systematically investigated using pristine Cu_3_P, ZnWO_4,_ and CZ NCs (CZ-5 to CZ-25) for the degradation of moxifloxacin (MOX), as presented in Fig. S1 (Electronic Supplementary Material).

### Fabrication of CZ-20 NC impregnated porous poly(EDGMA) monolith

For the synthesis of CZ-20 NC-embedded monolithic photocatalysts, 0.25 g of CZ-20 NC was ultrasonicated in a porogenic solvent (dry DMF) for 15 min. The resulting solution was equilibrated with 2.5 mmol of ethylene glycol dimethacrylate (EGDMA), serving as the monomer and crosslinker, along with 0.1 mmol of azobisisobutyronitrile (AIBN) as the free radical initiator. The reaction mixture was purged with N_2_ gas to remove dissolved oxygen and prevent radical scavenging, and the polymerization process was allowed to proceed for 24 h at 60 °C. After the reaction was complete, the unreacted oligomers were removed by washing with a hexane-water mixture. The resulting new-age photoactive NCs embedded poly(EDGMA) monolithic photocatalyst, i.e., CZ-20@PEM, was vacuum-dried overnight at 40 °C. A poly(EGDMA) monolith (PEM) was also synthesized without CZ-20 NC to compare the photocatalytic activity. The schematic representation of the synthesis of Cu_3_P, ZnWO_4_, CZ nanocomposites, and the CZ-20@PEM photocatalyst is shown in Scheme 1 (Electronic Supplementary Material).

### Design of photocatalytic experiments

Photocatalytic degradation studies targeting MOX removal were conducted using both CZ NCs and the CZ-20@PEM composite as catalysts within an annular photoreactor. The setup featured a tungsten filament lamp (240 W/m^2^) as the source of visible light, while a double-walled water jacket maintained ambient temperature regulation. In the trial experiments, 50 mg of the photocatalyst was dispersed in a 10 ppm MOX solution, which was stored in quartz tubes with an overall volume of 100 mL. Before photocatalytic experiments, the MOX solutions containing the dispersed photocatalyst were maintained in the dark for 30 min at the optimized pH to establish adsorption–desorption equilibrium between the photocatalyst surface and the pollutant molecules. To examine the interaction between moxifloxacin molecules and the surface of the CZ-20@PEM photocatalyst, adsorption–desorption and adsorption isotherm studies were performed, and the corresponding results are provided in Fig. S2(a&b) (*Electronic Supplementary Material*). To evaluate the photocatalytic degradation efficiency of MOX under visible light, absorbance at 295 nm was measured using a UV–Vis spectrophotometer. The subsequent formation of photoproducts and the associated degradation pathway were characterized through high-resolution mass spectrometry (HR-MS).

## Results and discussion

### Characterization of Cu_3_P–ZnWO_4_ and CZ-20/ PEM photocatalyst

#### Phase composition, surface topography, and structure morphology analysis

**Fig. 1:**
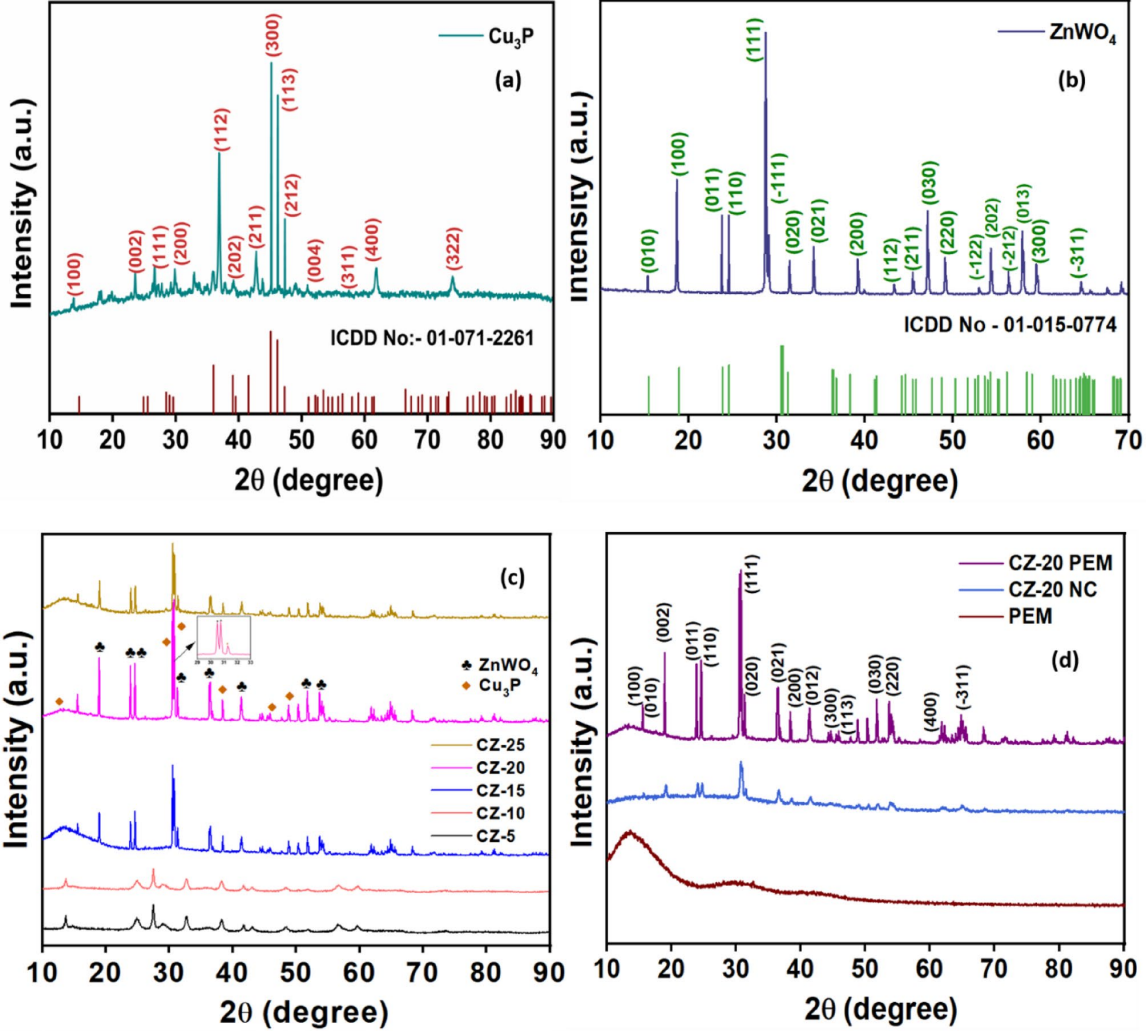
p-XRD pattern of (**a**) pristine Cu_3_P, (**b**) pristine ZnWO_4_, (**c**) CZ NCs, and (**d**) pristine PEM template CZ-20 NC and CZ-20@PEM photocatalyst.

Powder X-ray diffraction (p-XRD) analysis was conducted to evaluate the crystallinity and phase purity of the synthesized photocatalyst materials (Fig. 1). The p-XRD pattern of Cu_3_P (Fig. 1a) confirmed the formation of hexagonal phase (ICDD Card No. 71-2261), with characteristic diffraction peaks (2θ) at 14.6°, 24.3°, 28.4°, 29.5°, 36.0°, 39.3°, 41.5°, 45.1°, 46.3°, 47.3°, 51.2°, 59.7°, 61.7°, and 73.8° indexed to the (100), (002), (111), (200), (112), (202), (211), (300), (113), (212), (004), (311), (400), and (322) miller planes, respectively. (Fig. 1b) depicts the p-XRD pattern of ZnWO_4_, confirming its monoclinic phase with a wolframite structure through diffraction peaks (2θ) at 15.5, 18.9, 23.7, 24.5, 28.7, 29.2, 31.5, 34.2, 38.5, 44.3, 44.7, 47.6, 50.2, 52.8, 54.3, 56.4, 58.1, 59.3, and 64.6. These observed diffraction peaks were assigned to the crystal planes (010), (100), (011), (110), (111), (-111), (020), (021), (200), (112), (211), (030), (220), (-122), (202), (-212), (013), (300), and (-311) miller planes, respectively (ICDD No. 15-0774). The XRD patterns for Cu_3_P and ZnWO_4_ did not reveal any discernible secondary phases or impurity peaks, indicating single-phase crystallinity with high purity. Figure 1c represents the diffractograms of CZ NCs, which showed peaks characteristic of the hexagonal phase of Cu_3_P and the monoclinic phase of ZnWO_4_ to confirm the heterostructure formation. The diffraction patterns of CZ NCs indicated that, despite a slight reduction in the peak intensities, the fundamental hexagonal phase of Cu_3_P remained unchanged after heterojunction formation with ZnWO_4_. In the p-XRD pattern of CZ-5 and CZ-10 NCs, the characteristic peaks of ZnWO_4_ were distinctly visible, whereas the peaks for Cu_3_P were less prominent due to its low stoichiometry.

The absence of diffraction peaks corresponding to other impurities indicates the high purity and excellent crystallinity of CZ NC. The successful formation of CZ-20 NCs is evidenced by the presence of distinct crystalline diffraction peaks corresponding to both ZnWO_4_ (♣) and Cu_3_P (◆). These results offer clear evidence of the successful synthesis of the CZ NCs, confirming the incorporation of Cu_3_P into ZnWO_4_. In Fig. 1d, the bare PEM revealed the existence of an amorphous phase through a broad diffraction peak centered at 26.2° (2θ). In contrast, the p-XRD pattern of the CZ-20@PEM photocatalyst showed peaks characteristic of PEM and CZ-20 NC, thus confirming the effective integration of the crystalline CZ-20 NC within the amorphous PEM template. The distinctive crystalline diffraction peaks of CZ-20 were observed in the CZ-20@PEM photocatalyst, with slight variations in peak intensity and position, indicating the formation of a heterojunction interface between the ZnWO_4_ and Cu_3_P components.

**Fig. 2:**
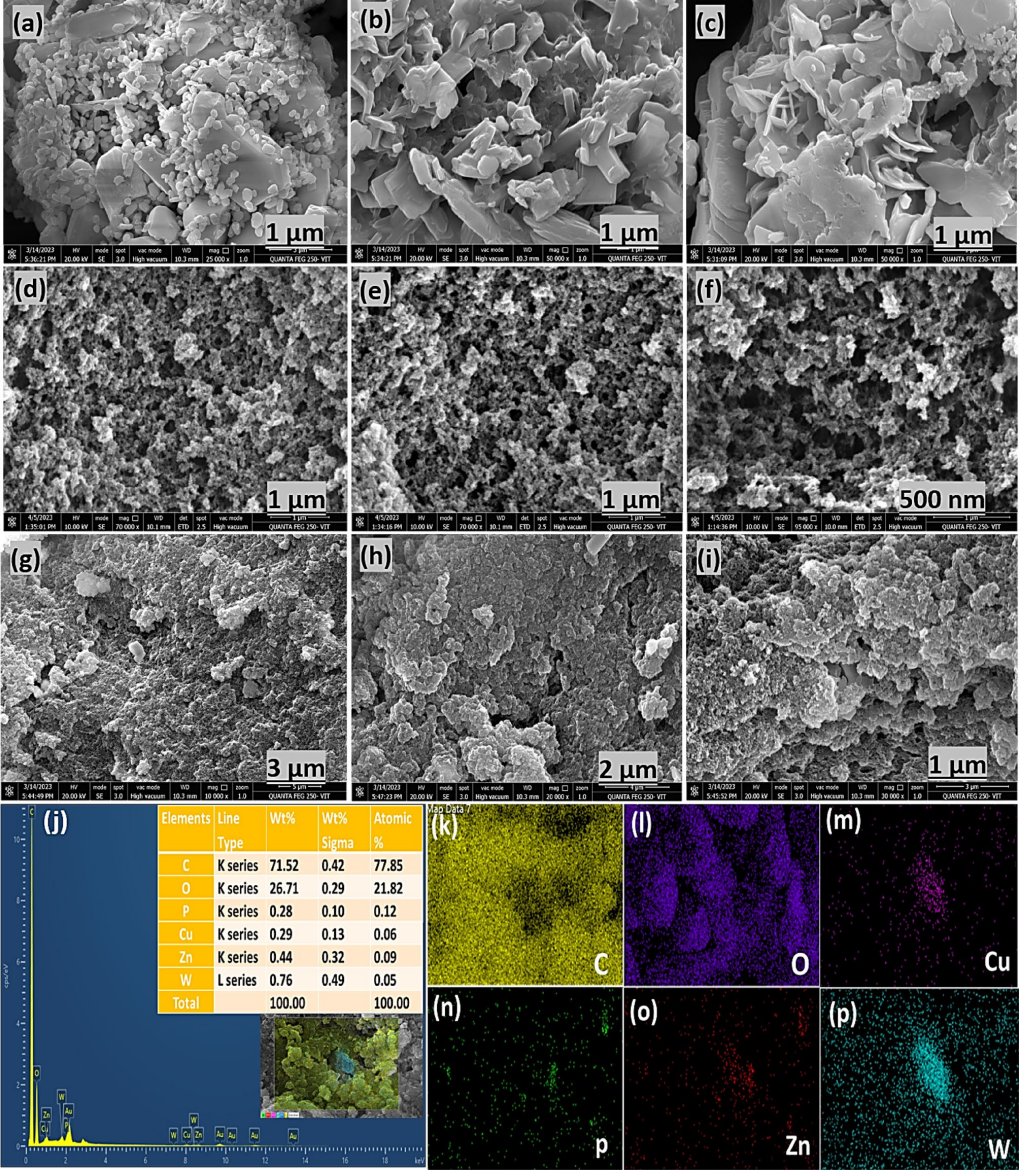
FE-SEM images of (**a–c**) CZ-20 NC, (**d–f**) pristine PEM, (**g–i**) CZ-20@PEM, (**j**) EDX spectra of CZ-20@PEM, and (**k–p**) Elemental mapping of CZ-20@PEM photocatalyst.

Surface and structural morphology analysis has been crucial for understanding the chemical events at the surface and the internal porous structural network of the synthesized photocatalyst, as examined using field-emission scanning electron microscopy (FE-SEM) and high-resolution transmission electron microscopy (HR-TEM). The FE-SEM images of pristine Cu_3_P and ZnWO_4_ nanoparticles have been presented in Fig. S3 (a-f) *(Electronic Supplementary Material)*. The FE-SEM images for CZ-20 NC revealed a homogeneous spindle-shaped Cu_3_P–ZnWO_4_ structure (Fig. 2a–c), indicating the presence of hexagonal-cross-sectioned nanoplate-shaped Cu_3_P NPs and granular morphology with a uniform distribution of ZnWO_4_ NPs. FE-SEM images of the pristine PEM (Fig. 2d–f) revealed a well-ordered, continuous, monolithic network with an interconnected macro- and mesoporous framework. The FE-SEM images at various resolutions (1 to 3 µm) of the CZ-20@PEM photocatalyst (Fig. 2g–i) revealed the presence of CZ-20 NC on the PEM template, with no significant changes to the CZ-20 NC morphology, showcasing the uniform distribution of CZ-20 NC across the surface of the PEM template.

The EDX analysis of pristine Cu_3_P, ZnWO_4,_ and PEM template (Fig. S3(g-l)) has been provided in the *Electronic Supplementary Material*. The EDX analysis (Fig. 2j) and elemental mapping (Fig. 2k–p) of CZ-20 PEM implied that all individual elements were present in the appropriate proportions. As shown in Fig. 2j, the element distribution image has been measured to confirm the presence/inclusion of Cu, P, Zn, W, C, and O in the CZ-20@PEM photocatalyst to ascertain the uniform integration of CZ-20 NC within the porous polymer template. The absence of impurity elements in the EDX data confirmed the purity of the CZ NC and photocatalyst. In the elemental distribution analysis (elemental mapping), Cu, P, Zn, W, and O species were distinguished by different colors to clearly illustrate their elemental distributions across the photocatalyst (Fig. 2k–p). The distribution patterns of Cu, P, Zn, W, C, and O species, as indicated by their assigned colors, confirm the uniform dispersion of CZ-20 NC within the porous poly(EGDMA) monolith.

**Fig. 3:**
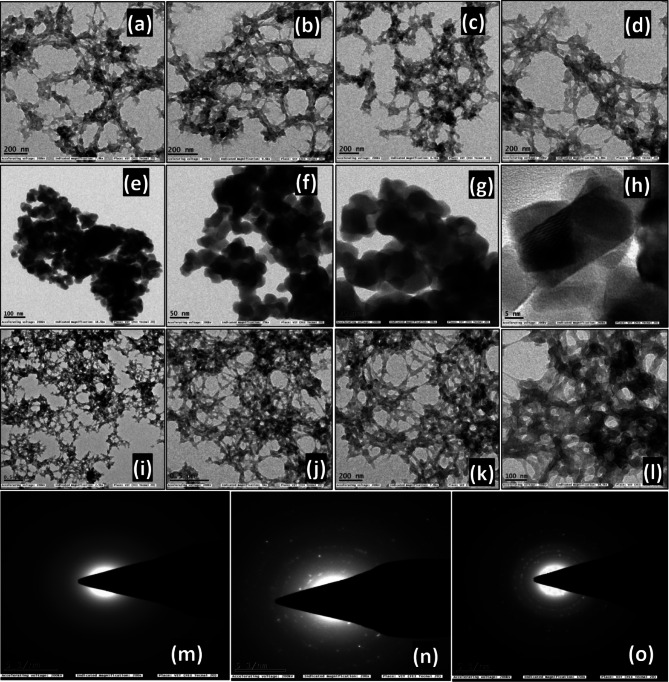
HR-TEM images of (**a–d**) pristine PEM, (**e–h**) CZ-20 NC, (**i–l**) CZ-20@PEM, and (**m–o**) SAED pattern of PEM, CZ-20 NC, and CZ-20@PEM.

HR-TEM analysis was performed to provide definitive evidence for CZ heterojunction formation. The TEM images of the PEM template (Fig. 3a–d) reveal structurally interlinked macroporous networks, with mesopore channels that facilitate fast transport and the generous adsorption of pollutant molecules onto the photoactive sites for photocatalytic degradation. TEM and HR-TEM images of the CZ NCs (Fig. 3e–h) confirmed the uniform distribution of Cu_3_P on the surface of ZnWO_4_. The crystallinity and phase identity of Cu_3_P and ZnWO_4_ were further validated through Fast Fourier Transform (FFT) analysis of the HR-TEM images, as presented in Fig. S4 *(Electronic Supplementary Material).* The FFT spots were indexed to the (hkl) planes of ZnWO_4_ and Cu_3_P, and inverse FFT filtering was used to highlight individual lattice fringes, which matched well with the ICDD values. The correlation between direct- and reciprocal-space imaging supports the reliability of phase identification, even though not all crystallites were imaged along a zone axis.

Further, the particle grain size was analyzed and tabulated in Table S2. (Electronic Supplementary Material). TEM images (Fig. 3i–l)) confirmed the effective distribution of CZ-20 nanocrystals (NCs) within the PEM matrix. The selected-area electron diffraction (SAED) pattern of pristine PEM (Fig. 3m) showed a lack of distinct diffraction spots, indicating an amorphous structure, in agreement with the p-XRD analysis. Conversely, the SAED pattern for CZ-20 NCs (Fig. 3n) exhibited bright diffraction spots arranged along rings, characteristic of a crystalline phase, and matching the specific lattice planes of Cu_3_P and ZnWO_4_, thus validating the heterojunction formation as observed in the p-XRD results. The SAED image of CZ-20@PEM (Fig. 3o) also revealed bright diffraction spots, affirming the presence of crystalline CZ-20 NCs embedded within the amorphous PEM host, and further confirming the successful synthesis of the composite photocatalyst.

#### Energy band gap and charge carrier separation studies

**Fig. 4:**
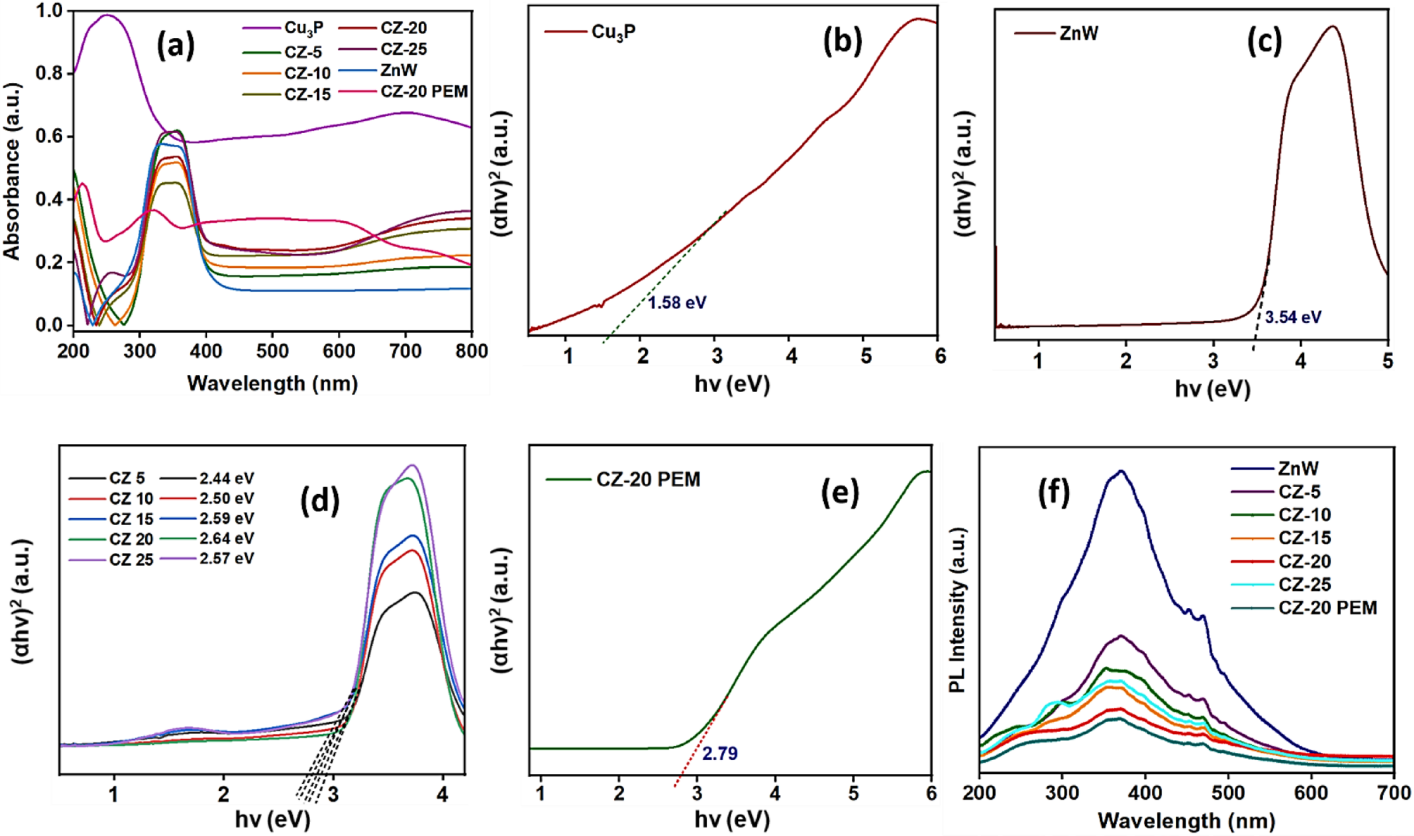
(**a**) UV-DRS data, (**b–e**) Tauc’s plot for Cu_3_P, ZnW, CZ NCs, and CZ-20@PEM photocatalyst, and (**f**) PL data of ZnW, CZ NCs, and CZ-20@PEM photocatalyst.

Photocatalytic performance was directly related to the photocatalyst’s ability to absorb visible light. Accordingly, the optical band gaps of the CZ NCs and CZ-20@PEM were determined via UV–Vis diffuse reflectance spectroscopy (DRS) to evaluate their visible light absorption capabilities, as depicted in Fig. 4. UV–Vis-DRS plots (Fig. 4a) for Cu_3_P demonstrated a strong absorption across the UV–Visible wavelength range. Consequently, ZnWO_4_ exhibited low absorbance in visible light, classifying it as a wide-bandgap photocatalyst with excitation limited to the UV spectrum, characterized by an optical absorption edge at 365 nm. The DRS data indicated that Cu_3_P responded to UV and visible light, whereas ZnWO_4_’s responsiveness was limited to the UV region, inducing electronic transitions. The DRS plot for CZ NCs and CZ-20@PEM revealed a noticeable redshift in the adsorption efficiency, with its absorption maxima in the visible light, suggesting that the stoichiometric inclusion of Cu_3_P to ZnWO_4_ enhanced the visible light absorption properties. The absorption wavelength measurements indicate that the CZ-20@PEM photocatalyst exhibited stronger visible-light absorption. The optical band gap energies were calculated using the classical Tauc’s plot, which revealed values of 1.58, 3.54, 2.64, and 2.79 eV for Cu_3_P, ZnWO_4_, CZ-20 NC, and CZ-20@PEM, respectively, as shown in Fig. 4b, e. The bandgap calculations revealed a substantial narrowing, with the introduction of Cu_3_P substantially reducing the band gap in CZ NCs. The observed data suggest a strong interaction between Cu_3_P and ZnWO_4_, which enhances the migration of e^−^/h^+^ pairs, thereby reducing the rate of charge-carrier recombination. The data showed that the CZ-20@PEM photocatalyst exhibits vigorous light-driven photocatalytic activity, with a significantly broader photo response, a crucial characteristic for visible-light-driven photocatalysis.

Photoluminescence spectroscopy (PL) was employed to investigate the transfer and separation of photogenerated charge carriers, which are crucial for generating reactive anionic and radical species for photocatalytic degradation, as illustrated in Fig. 4f. Generally, a low PL peak intensity corroborated the improved separation efficiency of photocatalysts-induced carriers observed with bare CZ-20 NC and CZ-20@PEM photocatalysts. The PL data revealed that pristine ZnWO_4_ exhibited the maximum peak intensity, indicating a faster recombination of e^−^/h^+^ pairs. Cu_3_P has been an exceptional semiconductor material, known for facilitating electron transport, a critical factor in enhancing photocatalyst activity. Notably, after forming a heterostructure NC between Cu_3_P and ZnWO_4_, the CZ-20 NC offered the least PL luminescence intensity among various CZ NCs, excluding CZ-20 PEM. Consequently, the low PL intensity observed in the CZ-20@PEM photocatalyst indicated efficient e^−^/h^+^ pair separation, thereby markedly enhancing photocatalytic activity. The enhanced visible-light absorption observed for CZ-20@PEM is attributed to the formation of heterostructures within the CZ NCs, leading to interstitial energy states that significantly reduce the recombination of photogenerated electron–hole pairs. The PEM template substantially increased the specific surface area, providing extraordinarily photoactive catalytic sites by extensively decorating CZ-20 NC.

Furthermore, to complement the PL analysis, electrochemical characterization of the synthesized catalysts was performed to confirm the effective separation of photogenerated charge carriers. The resulting electrical responses provide clear evidence of photoinduced charge transfer among the constituent materials. To further investigate the charge carrier dynamics, time-resolved photoluminescence (TrPL) measurements (Fig. S5) and electrochemical impedance spectroscopy (EIS) analyses (Nyquist plots, Fig. S6) were conducted for ZnWO_4_, CZ-5, CZ-10, CZ-15, CZ-20, CZ-25, and CZ-20@PEM. The detailed results are presented in the *Electronic Supplementary Material*. The combined PL, TrPL, and EIS investigations demonstrate that the CZ-20@PEM photocatalyst significantly enhances the separation efficiency of photoexcited charge carriers while effectively suppressing their recombination, a pivotal factor in its enhanced photocatalytic activity.

#### Surface area and pore dimension analysis

**Fig. 5:**
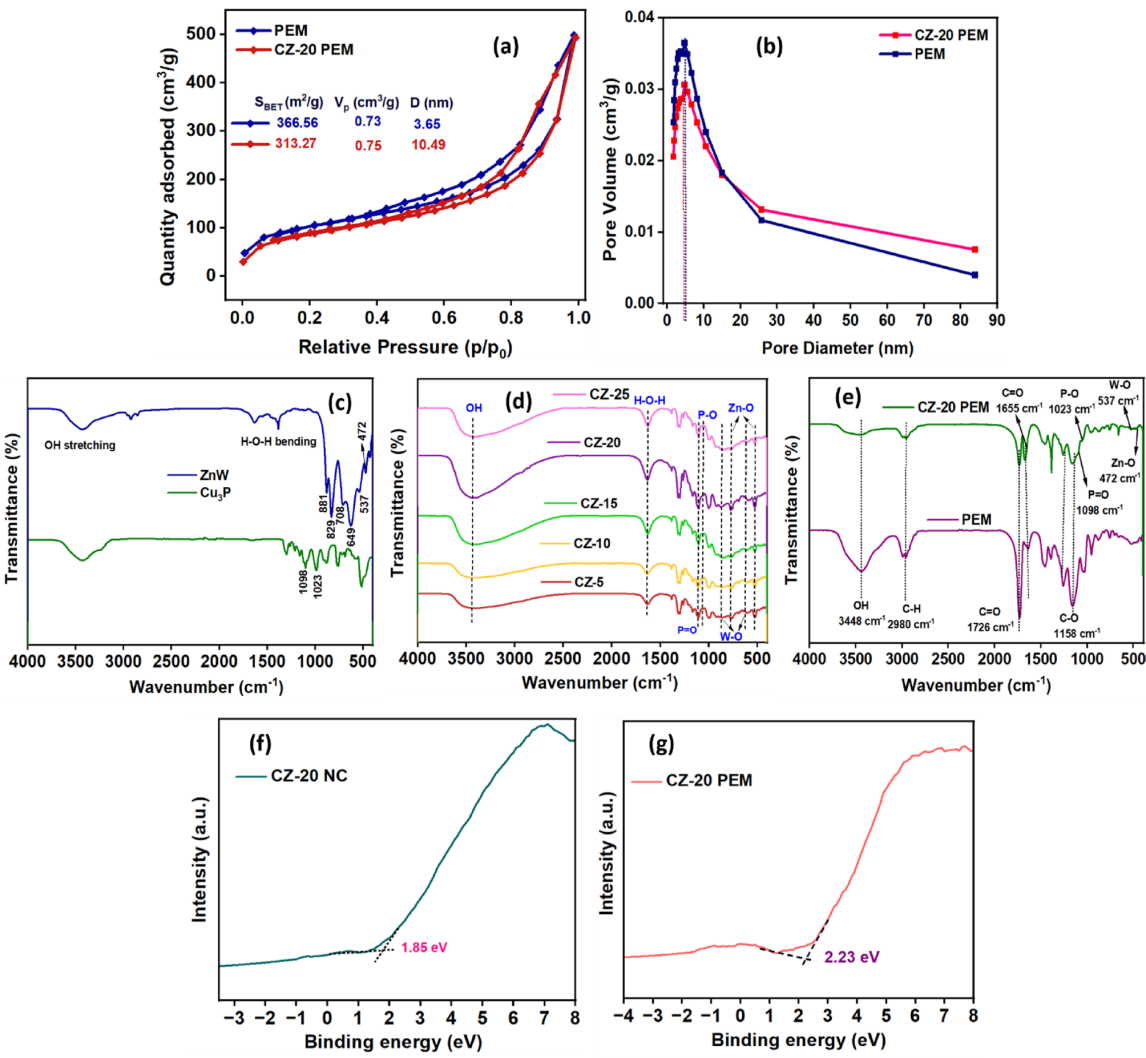
(**a**) Surface area analysis (BET), (**b**) pore size distribution analysis (BJH) of PEM and CZ-20@PEM. FTIR spectra of (**c**) pristine CuP and ZnW, (**d**) CZ NCs, (**e**) PEM, and CZ-20@PEM. VB-XPS analysis of (**f**) CZ-20 NC and (**g**) CZ-20@PEM.

The surface area and porosity characteristics play a crucial role in the photocatalytic efficacy of the photocatalyst in terms of the diffusion of pollutant molecules and their interaction with the photo-responsive sites, for which Brunauer‐Emmett‐Teller (BET) and Barrett-Joyner-Halenda (BJH) analysis were carried out for the bare PEM and CZ-20@PEM. Photocatalysts with a high surface area and porosity features ensure a higher adsorption capacity for pollutant molecules, thereby enhancing degradation efficiency. This occurs because adsorption facilitates efficient interaction between MOX pollutant molecules and the photocatalyst’s photoactive sites during photon irradiation. Consequently, achieving a high photodegradation rate necessitated an efficient adsorption process under optimal conditions. The adsorption–desorption isotherm profiles of both bare PEM and CZ-20@PEM photocatalyst display an H_1_ hysteresis loop characteristic of a type IV adsorption/desorption isotherm, confirming the presence of meso-/macro-pores (Fig. 5a). The isotherm plot revealed surface areas of 366.56 and 313.27 m^2^/g and pore volumes of 0.73 and 0.75 cm^3^/g for the PEM and CZ-20@PEM, respectively. The surface area and pore volume data suggested that the PEM template and CZ-20@PEM photocatalyst can exhibit high adsorption capacities for the pollutant molecules. The surface area, pore volume, and pore diameter data have been depicted in Table 1 and Fig. 5a. The data show a decrease in surface area for CZ-20@PEM relative to bare PEM, confirming the intercalation of microporous CZ-20 NC within the PEM template. From the BJH plot (Fig. 5b), the calculated pore size for bare PEM and CZ-20@PEM was 3.65 and 10.49 nm, respectively, to signify bimodal intertwined mesopore channels within the macroporous network for the synthesized template and photocatalyst materials. The high surface area and porosity of the CZ-20@PEM photocatalyst could potentially enhance visible-light-induced pollutant dissipation at the photoactive sites through diffusion and adsorption, and also improve surface-light interactions, electron mobility, and overall reactivity. Although BET surface area and BJH pore volume analysis were used to estimate adsorption capacity, the distribution, strength, and heterogeneity of adsorption sites were not fully captured by these parameters. To further investigate the adsorption behaviour, the adsorption substitution method was employed, and the resulting data were analysed using the Freundlich isotherm model. A strong correlation (R^2^) value of 0.98 indicated that adsorption occurred on the heterogeneous surface with multilayer coverage. This complementary analysis further confirmed that adsorption was governed not only by the physical surface characteristics but was also significantly influenced by the surface chemistry and electronic properties of the photocatalyst. These factors collectively enhanced visible-light absorption, charge-carrier separation and transfer, and ultimately improved the overall photocatalytic efficiency.

**Table 1:** Surface area, pore volume, and pore diameter data of PEM and CZ-20 PEM.

	S_BET_ (m^2^/g)	V_P_ (cm^2^/g)	D(nm)
PEM	366.56	0.73	3.65
CZ-20@PEM	313.27	0.75	10.49

#### Structural confirmation analysis

FT-IR analysis examined the chemical bonds and functional groups in the pristine Cu_3_P, ZnWO_4_, CZ NCs, bare PEM, and CZ-20@PEM materials, as presented in Fig. 5c–e. The FT-IR spectrum (Fig. 5c) for Cu_3_P revealed characteristic stretching vibrational peaks at 1023 and 1098 cm^−1^ of the Cu–P bond^26^. ZnWO_4_ displayed a wolframite crystal structure, characterized by vibrational peaks at 881 and 829 cm^−1^, which correspond to the W–O bonds in the WO_4_^2−^ units. Additional peaks observed at 649 and 708 cm^−1^ were associated with bending and stretching vibrations, reflecting the material’s high crystallinity and minimal defects. The peaks appearing at 537 and 472 cm^−1^ were assigned to the symmetrical and asymmetrical deformations of bridging oxygen atoms in Zn–O-W linkages, as well as to the W–O and Zn–O bonds within the WO_6_ and ZnO_6_^27^. In the FT-IR spectra of CZ NCs (Fig. 5d), the presence of both Cu_3_P and ZnWO_4_ was confirmed through characteristic stretching vibrations of Cu-P and Zn-W bonds, verifying the successful heterojunction formation between Cu_3_P and ZnWO_4_, resulting in the Cu_3_P–ZnWO_4_ heterostructure. Moreover, the intense peak at 3448 cm^−1^ was attributed to adsorbed water molecules.

In the FT-IR analysis of bare PEM (Fig. 5e), a prominent band at 3448 cm⁻^1^ was attributed to O–H stretching vibrations from moisture adsorbed on the surface. Peaks located at 1655 and 1726 cm^−1^ were linked to carbonyl (C=O) functionalities within the poly(EDGMA) backbone, while the peak at 1158 cm^−1^ was assigned to C–O stretching vibrations. Additionally, the 2980 cm^−1^ peak corresponded to C-H bond vibrations present in the PEM structure. For the CZ-20@PEM sample, the FT-IR spectrum displayed distinct features confirming the integration of Cu_3_P–ZnWO_4_ nanocrystals along with the characteristic bands of the PEM framework. This indicated the effective integration of CZ-20 nanocomposites within the PEM template without altering its structural integrity, in agreement with p-XRD results. Specifically, stretching peaks at 1023 and 1098 cm^−1^ were attributed to Cu_3_P, whereas peaks at 537 and 472 cm^−1^ corresponded to W–O and Zn–O vibrations in ZnWO_4_, thus confirming its dispersion within the PEM template and also the formation of the CZ-20@PEM photocatalyst.

#### Elemental species and oxidation state analysis

**Fig. 6:**
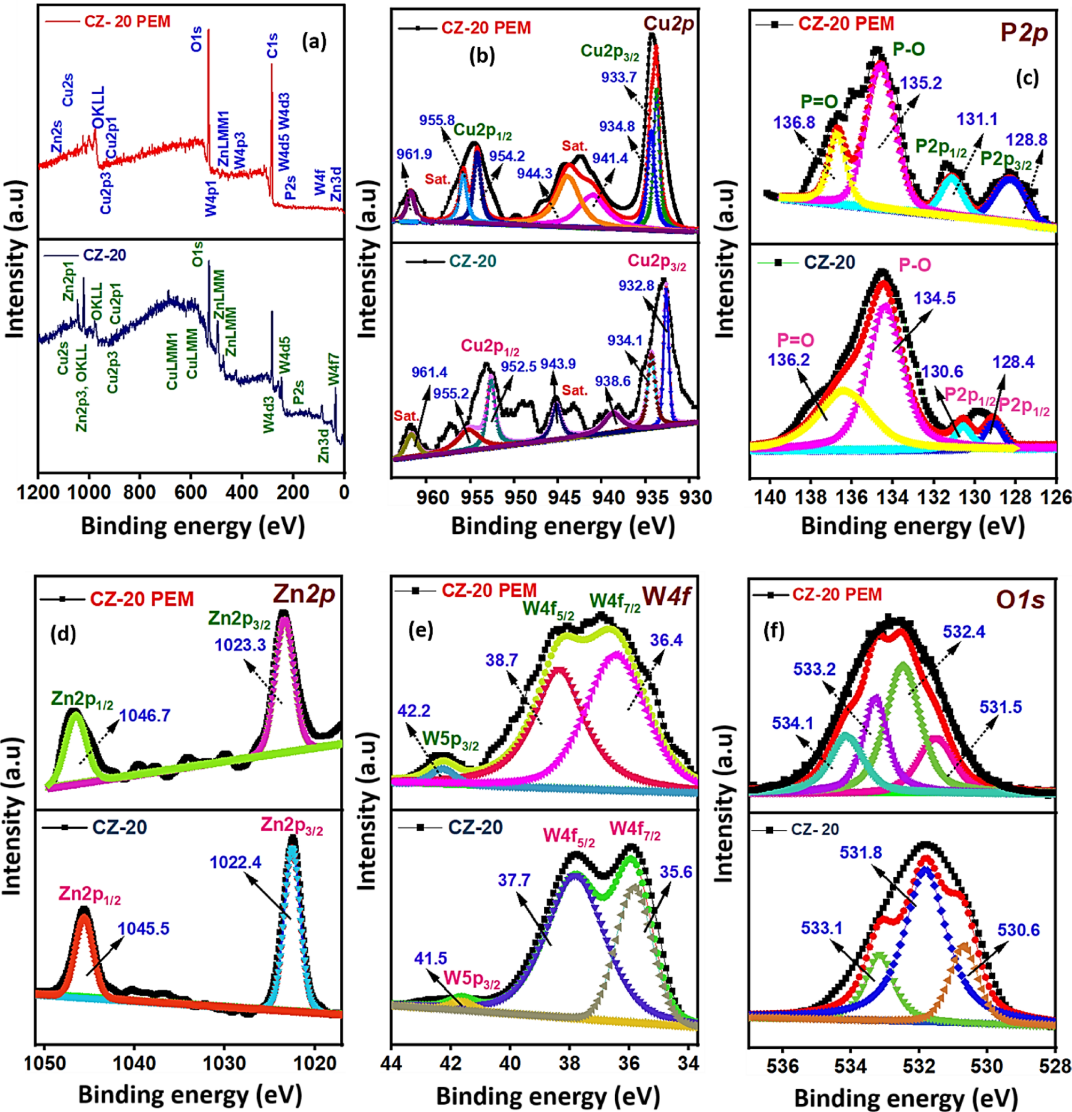
(**a**) XPS complete survey data and (**b**–**f**) deconvoluted XPS data of Cu*2p*, P*2p*, Zn*2p*, W*4f*, and O*1s* orbital states of CZ-20 NC and CZ-20@PEM photocatalyst.

The surface elemental composition and oxidation/electronic state of the CZ-20 NC and CZ-20@PEM photocatalysts have been investigated using X-ray photoelectron spectroscopy (XPS). The valence band (VB) positions of CZ-20 and CZ-20@PEM were determined using VB-XPS analysis (Fig. 5f, g). The study indicated that the CB and VB potentials for the CZ-20 NC were − 0.79 and + 1.85 eV, respectively. For the CZ-20@PEM photocatalyst, these values were − 0.26 and + 2.23 eV, respectively. The VB-XPS analysis provided an in-depth understanding of the photocatalysis mechanism during visible-light capture. The XPS analysis for the CZ-20 NC (Fig. 6a) confirmed the presence of Cu, P, Zn, W, and O. In contrast, the CZ-20@PEM photocatalyst exhibited Cu, P, Zn, W, C, and O. The deconvoluted high-resolution XPS spectra of CZ-20 and CZ-20 PEM signified the presence of Cu*2p*, P*2p*, Zn*2p*, W*4f*, and O*1s* states, as displayed in Fig. 6b–f. Specifically, the high-resolution XPS spectrum of Cu*2p* in Cu_3_P (Fig. 6b) can be deconvoluted into distinct peaks corresponding to the Cu*2p*_*1/2*_ and Cu*2p*_*3/2*_ energy states, along with three characteristic satellite peaks. The deconvoluted Cu*2p* spectra indicate the presence of Cu in multiple oxidation states, specifically Cu^+^ and Cu^2+^. The XPS spectrum exhibited peaks at 932.8 and 952.5 eV, agreeing with the Cu*2p*_*3/2*_ and Cu*2p*_*1/2*_ states of Cu^+^ in Cu_3_P, whereas the binding energies at 934.1 and 955.2 eV were assigned to the Cu*2p*_*3/2*_ and Cu*2p*_*1/2*_ states of Cu^2+^ in Cu_3_P. In the case of the CZ-20@PEM photocatalyst, the observed peaks at 933.7 and 954.2 eV were attributed to the Cu^+^*2p*_*3/2*_ and Cu^+^*2p*_*1/2*_ energy states in Cu_3_P, whereas the signals at 934.8 and 955.8 eV were associated with the Cu^2+^*2p*_*3/2*_ and Cu^2+^*2p*_*1/2*_ states, respectively. The binding energy peaks at 938.6, 943.9, and 961.4 eV for CZ-20 NC, and those at 941.4, 944.3, and 961.9 eV for the satellite peaks of CZ-20@PEM photocatalyst. These satellite peaks typically arise from the “shake-up” process, in which excess electrons are excited to higher-energy states^28^. Figure 6c presents the XPS P*2p* orbital state, exhibiting a characteristic doublet at 128.4 and 130.6 eV, which can be attributed to the P*2p*_*3/2*_ and P*2p*_*1/2*_ states for the CZ-20 NC, and for CZ-20@PEM, it was 128.8 and 131.1 eV, respectively. In addition, the peaks located at 134.5 and 136.2 eV for CZ-20 and peaks at 135.2 and 136.8 eV for CZ-PEM were attributed to P–O and P=O bonds, which were probably due to the surface oxidation of Cu_3_P after being exposed to air as a result of unavoidable sample processing.

The high-resolution XPS spectrum of Zn*2p* (Fig. 6d) for CZ-20 showed two distinct peaks at 1022.4 and 1045.5 eV, corresponding to the Zn*2p*_*3/2*_ and Zn*2p*_*1/2*_ states, respectively. The CZ-20@PEM showed characteristic peaks for the Zn 2p_3/2_ and Zn 2p_1/2_ states at 1023.3 and 1046.7 eV, respectively^29^. The W*4f* core level XPS spectrum of CZ-20 (Fig. 6e) was deconvoluted into a doublet with spin-orbital peaks at 35.6 eV and 37.7 eV corresponding to W*4f*_*7/2*_ and W*4f*_*5/2*_ states, respectively, along with an additional loss feature at 41.5 eV assigned to W*5p*_*3/2*_. These features confirm the presence of tungsten in the W^6+^ chemical state. For the CZ-20@PEM photocatalyst, the W*4f*_*7/2*_ and W4f_5/2_ peaks appear at 36.4 eV and 38.7 eV, with the W*5p*_*3/2*_ loss feature at 42.2 eV, reflecting a shift of approximately 0.7–1 eV to up-conversion binding energies. The shift suggested a change in electron density, likely due to oxygen vacancies^30,31^. This phenomenon was validated by deconvolving the O1s signal and curve-fitting (Fig. 6f), which revealed three distinct peaks at 530.6, 531.8, and 533.1 eV. These peaks were attributed to lattice oxygen in the Zn–O and W–O bonds of ZnWO_4_, as well as to surface chemisorbed oxygen in the case of CZ-20 NC. In CZ-20@PEM, the peaks at 531.5 and 532.4 were assigned to the Zn–O and W–O bonds, and the binding energy peaks at 533.2 and 534.1 eV correspond to the C–O–C and O–C=O skeletal groups of the PEM template^32^. A comparison of the XPS results from CZ-20 NC (Fig. S7), the CZ-20@PEM photocatalyst revealed higher binding energy shifts in Cu*2p*, Zn*2p*, W*4f*, P*2p*, and O*1s* orbital states to suggest the overlapping of electronic energy states for efficient charge carrier transfer/separation as a result of heterojunction formation and the intercalation of CZ NCs across the porous PEM template. Valence-band X-ray photoelectron spectroscopy (VB-XPS) analysis provided valuable insights into surface functional groups and chemical states, contributing significantly to understanding the electronic structure. The derived band-edge positions were used to construct the electronic band structure of the photocatalyst. Collectively, the results from the various characterization techniques not only validated the material’s structural integrity and stability but also facilitated a mechanism-driven interpretation of CZ-20@PEM photocatalytic behaviour, supporting its relevance for environmental remediation.

### Optimization of analytical parameters for enhanced photocatalytic activity

In evaluating the degradation of the target pollutant, it is essential to account for several analytical parameters, including solution pH, photocatalyst dosage, pollutant concentration, oxidizers/sensitizers, visible light intensity, scavengers, and the photocatalyst’s reusability and durability. The following subsections provide a detailed assessment of how these factors influence the photodegradation performance of the CZ-20@PEM photocatalyst toward the rapid and efficient removal of moxifloxacin.

#### Solution pH and photocatalyst dosage

**Fig. 7:**
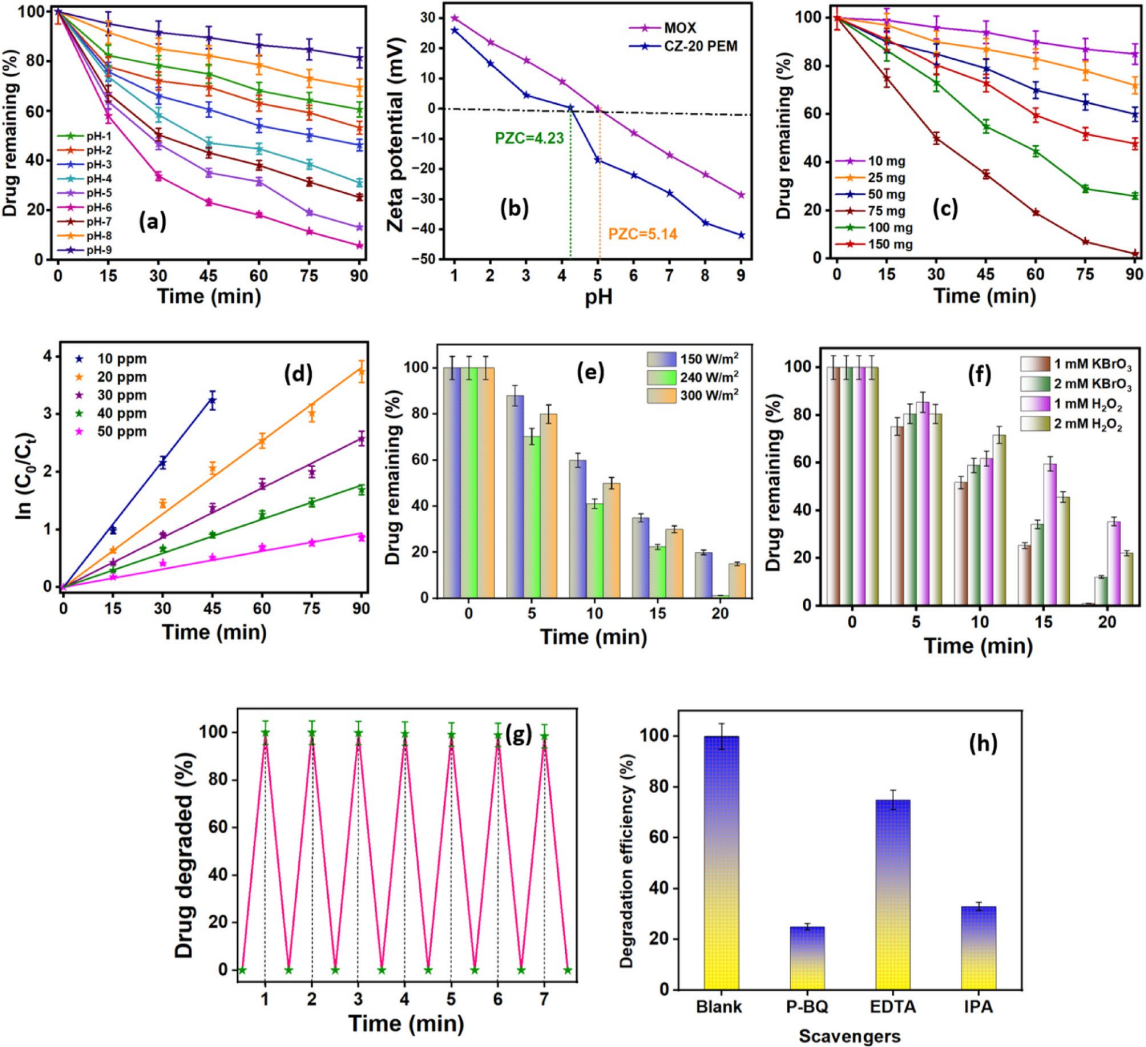
Effect of (**a**) solution pH *(RSD: 2.85)*, (**b**) zeta potential, (**c**) photocatalyst ratio *(RSD: 1.76)*, (**d**) initial pollutant concentration *(RSD: 1.34)*, (**e**) visible light intensity *(RSD: 1.50)*, (**f**) sensitizers/oxidizers *(RSD: 1.63)*, (**g**) reusability studies *(RSD: 3.48)* and (**h**) scavenger experiments *(RSD: 1.08)* on the photocatalytic process of MOX drug molecules using CZ-20@PEM photocatalyst.

Optimizing the solution pH for the dissipation of the target pollutant remained a critical factor, as the solution pH at which the photocatalyst is dispersed influences its surface charge, conduction/valence band edges, and NC aggregation. These modifications subsequently affected the adsorption of contaminants on the photocatalyst’s surface, altering the adsorbent’s surface charge characteristics and the ionic state of the pollutant molecules, thereby affecting the degradation kinetics and extent. In this study, the adsorption of the MOX antibiotic for eventual photocatalytic degradation was examined across a range of pH (2.0–9.0) with a 10 mg/L MOX concentration and a photocatalyst dosage of 0.005 g/L, as illustrated in Fig. 7a. The profile showed that the photocatalytic efficiency was maximum at pH 6.0 for the degradation of MOX molecules. It can be inferred that the surface properties, particularly the photocatalysts’ surface charge, significantly influence the degradation process. Hence, the pH at the point of zero charge (pH_pzc_) was determined for the MOX molecules and CZ-20@PEM photocatalyst to assess its surface charge by zeta potential analysis. The electrostatic interactions between the CZ-20@PEM and MOX at various pH conditions were analysed, as depicted in Fig. 7b. MOX exhibited an isoelectric point at pH 4.23, while the CZ-20@PEM photocatalyst had it at 5.14, which is consistent with the observed maximum photocatalytic efficiency within the pH range of 5.0–6.0. It was noted that MOX molecules, in their zwitterionic state, interacted optimally with CZ-20@PEM, particularly at pH 5.14, the isoelectric point. The reduced degradation efficiency at pH ≤ 4.0 can be ascribed to the positively charged surface of CZ-20@PEM, which induces electrostatic repulsion with the cationic and zwitterionic forms of MOX. Conversely, at pH ≥ 6.5, the predominance of negatively charged sites on the photocatalyst surface leads to strong electrostatic repulsion with the anionic form of MOX, thereby limiting its adsorption.

The quantity of photocatalysts significantly influences the photocatalytic degradation efficiency/rate, for which varying doses (10–150 mg) of CZ-20@PEM photocatalyst were dispersed in the reaction medium to investigate the extent of degradation/dissipation of MOX, as illustrated in Fig. 7c. It can be observed that variations in the photocatalyst dosage have both positive and negative impacts on the degradation kinetics. With increasing amounts of photocatalyst, the drug degradation rate increased due to a greater availability of photoactive sites that can effectively interact with incident photons, up to 75 mg. Consequently, more MOX molecules become adsorbed onto the photocatalyst surface due to increased particle density in the illuminated area. The results indicate that the degradation efficiency initially increases with increasing photocatalyst dosage, then levels off. However, above 75 mg, the higher catalyst loading increased the opacity of the MOX solution, reducing photon penetration into the medium and consequently diminishing photocatalytic degradation. In addition, surface area reduction due to particle agglomeration (particle–particle interactions) has been observed at higher dosages. The solution’s turbidity reduced the generation of charge carriers, thereby reducing the production of reactive oxygen species (ROS) via surface diffusion. Therefore, a dosage of 75 mg has been considered optimal and used for subsequent analyses.

#### MOX concentration and light intensity

The photocatalytic degradation efficiency of a given pollutant is primarily influenced by its intrinsic chemical and physical properties, as well as its initial concentration, which governs the degree of adsorption onto the photocatalyst surface. Adsorption of drug molecules onto the catalyst surface was found to be a critical step in facilitating effective degradation. Therefore, the pollutant’s starting concentration plays a key role in determining the extent of surface adsorption, ultimately impacting degradation performance. As shown in Fig. 7d, the degradation behaviour of MOX was evaluated across various initial concentrations (10–50 ppm) under optimized conditions of pH 6.0 and a photocatalyst loading of 75 mg. The profile indicated that the degradation kinetics at low MOX concentration were faster, eventually declining beyond 30 ppm. At a fixed photocatalyst dosage, the degradation rate decreased due to increasing drug concentration. This behaviour can be attributed to the fact that at lower initial concentrations, a higher proportion of drug molecules undergo photoexcitation and energy transfer, leading to an enhanced degradation rate. However, when the concentration exceeds 30 ppm, the efficiency decreases due to a shielding effect, where excess drug molecules obstruct incident photons from reaching the photocatalyst surface, thereby diminishing light absorption and reducing photocatalytic degradation performance. Furthermore, at high MOX concentrations, a significant number of pollutant molecules adsorb to the CZ-20@PEM surface, hindering the interaction of incident photons with the photocatalyst’s photon-responsive sites. This leads to a slower generation of reactive oxygen species, thereby diminishing the overall degradation efficiency. Additionally, using high concentrations of MOX led to extensive formation of MOX intermediates and photoproducts, which tend to accumulate near the photoactive sites, further blocking incoming photons from reaching them. However, despite the apparent reduction in the degradation efficiency, the amount of dissipated MOX molecules increased significantly with increasing MOX concentrations. The observation revealed that the proposed CZ-20@PEM demonstrated strong visible-light photocatalytic performance even at higher pollutant concentrations, but only for an extended duration.

The influence of light intensity remained a crucial factor in determining the quantity of incident photons available to interact with the photocatalyst and generate reactive species that facilitated degradation. Hence, the impact of light intensity on the photocatalytic degradation of MOX has been assessed through the photocatalytic performance of CZ-20@PEM, which was tested at various visible light intensities of 150, 240, and 300 W/m^2^ (using a tungsten filament lamp), as illustrated in Fig. 7e. It was evident that the quanta of energy required for the voluminous photoexcitation of the charge carriers from the photocatalyst valence band to the conduction band depended on the intensity of the visible light irradiation. The degradation pattern indicated that, for a 150 W/m^2^ visible light source, approximately 84% of the drug molecules were degraded within 1 h of irradiation, exceeding 99.0% of the MOX photocatalytic degradation at 240 and 300 W/m^2^. With increasing light intensity, the generation and separation of electron–hole pairs were markedly enhanced, leading to the formation of reactive oxygen species and thereby accelerating the photocatalytic degradation of MOX. It was inferred that light intensities above 240 W/m^2^ had no discernible effect on photocatalytic deterioration. The findings showed that the maximum photon flux required for excitation within a specific area of the photoactive sites increased with increasing irradiation intensity. Hence, the photocatalytic degradation efficiency did not vary significantly with further increases in light intensity. The results showed that energy conversion increased at the optimal light intensity, thereby minimizing thermal pollution and costs.

#### Electron acceptors, reusability, and practical utility

Introducing electron acceptors into the photocatalytic process can reduce charge-carrier recombination and promote ROS formation, thereby enhancing process efficacy. Accordingly, this study investigates the effect of oxidizing agents on photocatalytic degradation, using KBrO_3_ and H_2_O_2_ at concentrations of 1.0–2.0 mM. These oxidizing agents act as electron acceptors in the photocatalytic degradation of MOX molecules. Figure 7f revealed that under optimal experimental conditions (pH 6.0, 75 mg of photocatalyst, 30 ppm of MOX, and 240 W/m^2^ tungsten lamp), the use of KBrO_3_ and H_2_O_2_ significantly improved the generation of reactive oxygen species, which accelerated the breakdown of target MOX molecules by mitigating the recombination or quenching effects. The effectiveness of the photocatalytic reaction was primarily dependent on the separation of e^−^/h^+^, which was efficiently achieved with KBrO_3_, which accepts the photogenerated electrons. Similarly, the stoichiometric addition of H_2_O_2_ increased the degradation rate by generating reactive hydroxyl radicals that facilitated MOX dissipation. However, a decline in efficiency can occur at a higher concentration of 2 mM KBrO_3_ as the BrO_3_^−^ concentration increases, due to competitive adsorption at the catalyst’s active sites. As the concentration of BrO_3_^−^ increases, competition for the limited active sites intensifies, reducing the probability that individual molecules interact with the photocatalyst and resulting in lower overall degradation efficiency. In the case of H_2_O_2_, it had a negligible effect on photocatalysis at < 1.0 mM, but at 1.0 mM, an enhanced degradation efficiency was observed, which declined above 1.0 mM. The inhibition can be attributed to surface modifications of CZ-20@PEM induced by H_2_O_2_ adsorption, which lead to the scavenging of photogenerated holes and to interactions with hydroxyl radicals. While H_2_O_2_ can enhance photodegradation by acting as an electron scavenger and promoting the generation of hydroxyl radicals, at higher concentrations it may hinder the process by facilitating recombination with ^•^OH radicals or photogenerated holes (h^+^), thereby reducing efficiency. Consequently, with 1.0 mM KBrO_3_ as the oxidizer, ≥ 99.4% MOX degradation was achieved within 20 min of photocatalysis using a 240 W/m^2^ visible light source.

The durability and stability of the synthesized CZ-20@PEM photocatalyst have been crucial parameters for evaluating its practical application. To assess this, recovery and reusability experiments were carried out, as shown in Fig. 7g. Stability studies of the photocatalyst demonstrated sustained performance of the CZ-20@PEM in dissipating MOX after repeated use. The photocatalyst was recovered by simple filtration, followed by thorough rinsing with deionized water and ethanol to prepare a fresh MOX solution for the next photocatalytic cycle. The recycled CZ-20@PEM catalyst was reused for up to seven cycles under similar conditions without any change in photocatalytic performance. The p-XRD patterns were compared to evaluate the stability of the CZ-20@PEM photocatalyst after seven reuse cycles, as depicted in Fig. S8(a) (*Electronic Supplementary Material*). The diffraction pattern remained unchanged even after several cycles, demonstrating the excellent stability of the CZ-20@PEM photocatalyst’s internal structure. Furthermore, the BEJ & BJH analysis (Fig. S8(b&c)) of the reused photocatalyst after seven reuse cycles showed a slight decline in surface area, from 313.27 to 296.42 m^2^/g, and in the average pore size, from 10.49 to 2.82 nm. The notable decrease in performance indicated that the CZ-20@PEM catalyst has undergone structural changes after several cycles of reuse. The reduction in pore size and surface area can be attributed to factors such as partial pore blockage by residual organic byproducts or intermediates from degradation, slight compaction of the porous framework due to repeated operational stress, and pore fouling caused by adsorption. These modifications could affect mass transport and active-site accessibility, thereby affecting the overall photocatalytic performance over time. The observed data confirmed that the CZ-20@PEM has undergone measurable changes in its pore structure across multiple cycles, underscoring the need to assess long-term stability and regeneration methods to sustain prolonged photocatalytic performance.

The FE-SEM-EDX line profile analysis for the CZ-20@PEM photocatalyst after seven reuse cycles was conducted to evaluate its structural and compositional stability, as shown in Fig. S8(d) (*Electronic Supplementary Material*). A slight variation in the EDX elemental percentages was observed between the fresh and reused samples. This difference can be attributed to several factors, which include surface leaching or partial loss of active metal species Cu, Zn, or W during repeated photocatalytic cycles, adsorption of reaction intermediates or degradation products on the catalyst surface, which may alter the detected elemental ratios, and surface restructuring or minor morphological changes induced by repeated irradiation and redox reactions during photocatalytic operation. These effects collectively contributed to slight deviations in the elemental composition of the reused photocatalyst. However, the overall distribution pattern remained relatively uniform, indicating good structural stability of CZ-20@PEM after multiple reuse cycles. The EDX line-scan analysis was employed to examine the spatial distribution of elements along a predefined path on the material’s surface. This technique provides detailed insights into the elemental composition across the scanned region, enabling assessment of the uniformity or possible localized variations in elemental distribution. The generated line profiles depicted the relative abundance of C, O, Cu, P, Zn, and W as a function of position along the selected scan line, thereby visualizing the spatial distribution of each element in the CZ-20@PEM photocatalyst. The corresponding X-ray signal intensities for these elements were plotted against the scanning distance, illustrating both elemental uniformity and potential compositional heterogeneity across the photocatalyst surface. The photocatalytic performance of the CZ-20@PEM catalyst for the degradation of MOX was further assessed using both synthetic and real water samples, as detailed in the *Electronic Supplementary Material* (Fig. S9).

#### Scavenging experiments and photocatalysis mechanism

Identifying the reactive species involved in the photocatalytic degradation process proved imperative for understanding and constructing a potential charge-transfer pathway, ultimately leading to a more precise elucidation of the photocatalytic degradation mechanism. Scavenging experiments were performed to elucidate the roles of reactive species, employing specific scavengers such as p-benzoquinone (p-BQ) for ·O_2_^−^, isopropyl alcohol (IPA) for ·OH, and ethylenediaminetetraacetic acid (EDTA) for h^+^. The corresponding results are illustrated in Fig. 7h. The photocatalytic effectiveness of CZ-20@PEM was barely affected by p-BQ, suggesting that ·O_2_^−^ was not as crucial in the MOX breakdown process. In contrast, adding IPA decreased photocatalytic performance, implying that ·OH radicals were a key species in the decomposition of MOX molecules into their photoproducts. In the case of EDTA, the most pronounced quenching effect demonstrated that the holes (h^+^) are also major reactive species leading the photocatalytic degradation of MOX.

Based on the identification of the reactive species involved in the photocatalytic process, determining the valence/conduction band positions and constructing an optimal energy band structure offers crucial insights into the redox potential of the valence and conduction bands. The redox potential energy diagram helps assess whether the Cu_3_P–ZnWO_4_ heterojunction has compatible band structures that facilitate efficient charge transfer and generate reactive species for MOX dissipation. Based on the VB-XPS analysis and the outcomes of the scavenger experiments, a plausible photocatalytic mechanism has been proposed for the CZ-20 NC and CZ-20@PEM photocatalyst. The visible-light-induced photocatalytic behaviour of CZ-20@PEM was closely linked to its band structure, underscoring the importance of considering the band energies of the semiconductors involved. The valence-band (VB) positions of CZ-20 NC and CZ-20@PEM were experimentally determined by VB-XPS, providing direct insight into their electronic structures. As the conduction band (CB) edge cannot be directly determined from XPS, it was calculated theoretically using the measured VB values in conjunction with established Eqs. (1 & 2). The CB and VB band edge potentials of Cu_3_P and ZnWO_4_ were estimated using Mulliken electronegativity theory, as described by the following equations:

1 $$E_{VB} = X - E_{e} + 0.5 (E_{g} )$$

2 $$E_{CB} = E_{VB } - E_{g}$$

**Fig. 8:**
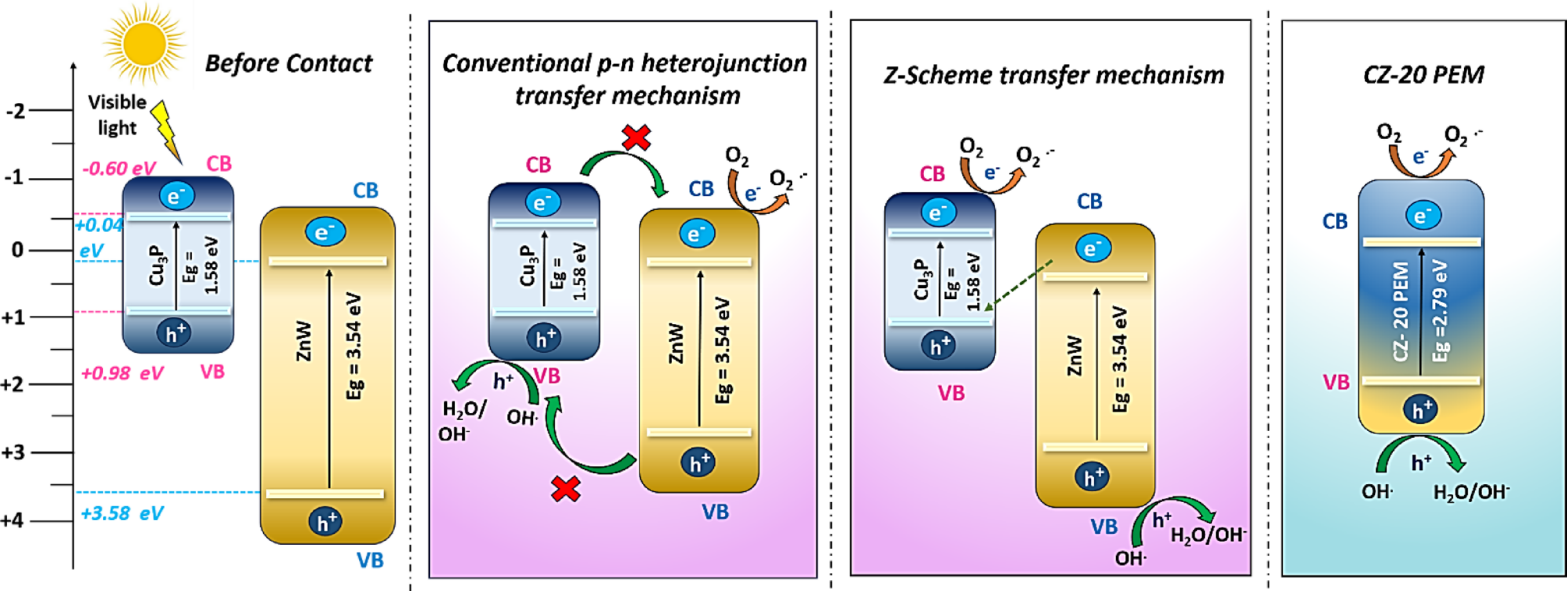
Visible-light-induced photocatalytic mechanism for the heterostructured CZ-20 NC and CZ-20@PEM.

Here, *X* represents the material’s electronegativity, defined as the geometric mean of the electronegativities of its constituent atoms. *E*_*e*_ represents free electron energy on the hydrogen scale (approximately 4.5 eV), while *E*_*g*_ corresponds to the band gap energy of Cu_3_P and ZnWO_4_. *E*_*VB*_ and *E*_*CB*_ refer to the VB and CB edges, respectively. The band potential calculations illustrated the band diagram of the CZ-20@PEM heterojunction, including the proposed charge transfer pathway and the alignment of the pristine Cu_3_P and ZnWO_4_ energy bands that led to the narrowing of the fermi level for charge carrier transfer/separation during visible light irradiation, as depicted in Fig. 8. Cu_3_P has been a predominant photoactive material under visible light illumination that has been the major factor towards optimal band gap narrowing through the formation of intermediate energy states with ZnWO_4_, which however was limited by its ability to undergo photoexcitation only in the UV region of the solar spectrum.

The valence band (E_VB_) and conduction band (E_CB_) positions of Cu_3_P were determined to be + 0.98 eV and − 0.6 eV, respectively, while those of ZnWO_4_ were calculated as + 3.54 eV and + 0.04 eV. From these values, the energy band structures of Cu_3_P and ZnWO_4_ were derived, indicating that both the VB and CB potentials of ZnWO_4_ were lower than those of Cu_3_P. The band alignment stated the formation of a Z-scheme heterojunction between Cu_3_P and ZnWO_4_, facilitating efficient spatial separation of photogenerated electron–hole pairs under visible-light irradiation and thereby enhancing photocatalytic performance. Interfacing of Cu_3_P and ZnWO_4_ involves the transfer of photogenerated electrons from the more positive CB state of ZnWO_4_ to the more negative VB state of Cu_3_P, driven by differences in Fermi energy levels, to reach equilibrium. The resulting built-in electric field, arising from this Fermi level alignment, can accelerate the recombination of photogenerated electron–hole pairs in a controlled manner, thereby improving overall photocatalytic efficiency^33^. Concurrently, the photogenerated electrons accumulated in the CB state of Cu_3_P, and the photogenerated holes accumulated in the VB of ZnWO_4_ due to the higher CB and lower VB edge potential of Cu_3_P and ZnWO_4,_ respectively, resulting in a heterostructure of higher reduction and oxidation potential. Besides, the VB potential of Cu_3_P was lower than the oxidation potential of hydroxyl radicals (E°(·OH/H_2_O) + 2.27 eV), which prevented the photogenerated holes in Cu_3_P from producing ·OH radicals through water splitting^34^. Photogenerated holes can directly degrade organic pollutants, as confirmed by scavenger experiments using EDTA. The formation of the ZnWO_4_-Cu_3_P heterojunction induces a built-in electric field, promoting electron transfer within the heterostructure and leading to hole accumulation. This process contributes to band bending and effective narrowing of the band gap, as illustrated in Fig. 8. Unlike a conventional type-II heterojunction, this system cannot be classified as such, since the VB potential of ZnWO_4_ (+ 3.54 eV) was significantly higher than the H_2_O/·OH potential (+ 2.27 eV), allowing the generation of hydroxyl radicals. Although a Type II heterojunction can facilitate spatial separation of photogenerated charge carriers by enabling electrons and holes to migrate in opposite directions across the interface, this configuration was likely to compromise the redox capability of the system. However, in the proposed Z-scheme pathway, electrons in the CB of Cu_3_P reduce O_2_ to form superoxide radicals (·O_2_^−^), while holes in the VB of ZnWO_4_ oxidize with H_2_O/OH^−^ to produce ·OH. This configuration effectively inhibited charge recombination, thereby promoting charge-carrier separation and boosting photocatalytic activity. Scavenger experiments further demonstrated that ·OH radicals, generated by hole oxidation, played a more significant role in pollutant degradation than ·O_2_^−^ radicals. These results underscore the pivotal roles of both photogenerated electrons and holes, demonstrating that the improved photocatalytic performance is attributable to the formation of a direct Z-scheme heterojunction between Cu_3_P and ZnWO_4_.

The superior visible-light photocatalytic performance of the proposed CZ-20@PEM photocatalyst has been compared with literature reports, as tabulated in Table 2^35–43^. The comparison data revealed that few reported photocatalysts achieved optimal performance only at pH values impractical for real-world applications. Moreover, although near-complete removal of MOX has been reported in some systems, it typically necessitated either excessively high photocatalyst dosages or prolonged irradiation times. In contrast, the proposed CZ-20@PEM photocatalyst demonstrated remarkably high photocatalytic activity for MOX degradation under visible-light irradiation, outperforming most previously reported photocatalysts.

**Table 2:** Literature comparison of various heterostructure catalysts with the proposed photocatalyst for the photocatalytic degradation of MOX drug.

S. No	Photocatalyst Material	Illumination Conditions	Catalyst Dosage (g)	pH	Degradation (%)	MOX (ppm)	Degradation Time (min)	Reusability Cycles	References
1	Ce_2_(WO_4_)_3_@ g-C_3_N_4_)	150 W/cm^2^ Tungsten lamp	0.05	–	94.1	10	60	5	^35^
2	g-C_3_N_4_ (GCN) with methoxybenzoyl	300 W Xe lamp	1	5.5	80	20	30	5	^36^
3	Co_3_O_4_/BiVO_4_	Natural Sunlight	0.05	–	68.3	10	160	5	^37^
4	Bio Co-FeMnO_x_	300 W Xe lamp	0.12	7–9	95	20	100	5	^38^
5	(NiFe-LDH/rGO)	10 W LED lamp	1	8	90.4	20	60	5	^39^
6	CuFeS_2_/MXene	300 W Xe lamp	0.12	5.8	99.1	2	40	7	^40^
8	V-MnO_2_	100 W/cm^2^ Tungsten lamp	0.01	3	79.5	20	300	3	^41^
9	SnO_2_/Cu_2_O	500 W Xenon lamp (λ ≥ 420 nm)	0.05		86.4	20	140	5	^42^
10	CoMn@CNT	–	0.03	5	89.7	10	30	4	^43^
11	CZ-20@PEM	240 W/m^2^ Tungsten lamp	0.075	6	≥ 99.4	30	20	7	Present Work

#### Photocatalytic degradation pathway of MOX

The HR-MS analysis has been used to identify the photoproducts generated by MOX during visible-light-induced photocatalysis of CZ-20@PEM under optimized conditions. The plausible photoproducts and intermediates pathway of MOX leading to mineralization revealed three distinct degradation processes: decarboxylation, decyclopropyl, and defluorination. The predominant intermediates of the MOX drug included the cyclopropyl-eliminated (M2), decarboxylated (M1), and defluorinated products (M6). In *pathway 1*, the intermediate M6 (*m/z* 301) was a defluorination product of MOX, leading to the formation of intermediates M7 (*m/z* 279), M8 (*m/z* 274), M10 (*m/z* 256), and M11 (*m/z* 254). In *pathway 2*, the M1 (*m/z* 384) decarboxylation photoproduct of MOX paved the route for the intermediate M3 (*m/z* 347) and M5 (*m/z* 317). In *pathway 3*, the decyclopropyl product of MOX, i.e., M2 (*m/z* 362), led to the formation of M4 (*m/z* 319) and M9 (*m/z* 273). A comparative analysis was performed between systems with and without the photocatalyst to highlight its role in the degradation process. The HRMS data for the non-photocatalyzed MOX drug and the identified photoproducts/intermediates of the photocatalyzed samples, along with the possible degradation pathway from the identified photoproducts, have been depicted in Fig. S10 & S11(a-c) and Scheme S2 (*Electronic Supplementary Material*). Under visible light photocatalytic degradation, MOX drug molecules were found to undergo a series of oxidative transformations rather than direct mineralization. In the initial stages, the heterocyclic side chain was primarily attacked by hydroxyl radicals and photogenerated holes, leading to hydroxylation, N-oxidation, N-dealkylation, and subsequent ring-opening reactions of the piperazine/pyrrolidinyl group. HRMS analyses revealed that hydroxylated, N-dealkylated, and ring-opened MOX derivatives were consistently degraded through photocatalysis. Upon prolonged irradiation, oxidation progressively affected the quinolone structure, leading to the formation of hydroxylated and decarboxylated compounds, which ultimately underwent aromatic ring cleavage to yield smaller intermediates. The HR-MS data confirmed the progressive degradation of MOX molecules, ultimately leading to mineralization, thereby ensuring effective decontamination.

## Conclusion

In this study, an innovative Z-Scheme Cu_3_P–ZnWO_4_ heterostructure NC has been synthesized and has been voluminously/homogeneously decorated onto a meso-/macro-pore poly(EGDMA) monolithic template by bulk polymerization technique. The synthesis of CZ NCs and the fabrication of the CZ-20@PEM photocatalyst were validated through various electron microscopy, diffraction, and spectroscopic methods, ensuring a comprehensive understanding of the photocatalyst’s physical, chemical, and electronic properties. Surface area and porosity measurements, derived from BET and BJH analyses, were correlated with the material’s pollutant adsorption and light interaction capabilities. The structural characteristics, including crystallinity, morphology, and particle size distributions, were confirmed via p-XRD, FE-SEM-EDAX, and HR-TEM-SAED techniques. Additionally, the identification of surface functional groups and chemical states of elements via VB-XPS provided valuable insights into the electronic structure, enabling predictions of charge-transfer mechanisms. Optical and electrochemical evaluations using UV–Vis-DRS, PL, TrPL, and EIS were performed to determine the band gap and explore charge-transfer dynamics. The photocatalytic performance of CZ-20@PEM under visible light was evaluated by its ability to degrade MOX molecules. Under optimized conditions (pH 6.0, 75 mg catalyst, 30 ppm MOX, 1.0 mM KBrO_3_, and 240 W/m^2^ visible light intensity), the photocatalyst achieved an impressive 99.4% degradation rate. The formation of a Z-scheme Cu_3_P–ZnWO_4_ heterojunction facilitated efficient charge transfer, enhancing photocatalytic activity. Compared with other binary and ternary systems, the CZ-20@PEM photocatalyst demonstrated higher degradation activity, stability, and reusability, highlighting its potential for wastewater treatment and environmental remediation. This work reports the first study of a Cu_3_P–ZnWO_4_ heterojunction, utilizing a novel strategy to decorate CZ NCs onto a translucent polymer monolith for the efficient degradation of pharmaceutical contaminants in water. The proposed CZ-20@PEM photocatalyst offers a cost-effective, eco-sustainable solution for heterogeneous photocatalysis. Moving forward, we aim to bridge the gap between laboratory-scale research and real-world applications, promoting practical solutions in photocatalytic water purification.

## Supplementary Information

Below is the link to the electronic supplementary material.

Supplementary Material 1

## Data Availability

The authors declare that the data supporting the findings of this study are available within the paper and its Supplementary Information files.
