# Supplementary material for: Engineered Cu3P–ZnWO4 heterojunction integrated with porous polymer monolithic template for enhanced photocatalytic degradation of organic pollutants
Source: Sci Rep. 2025 Dec 1;16:389. doi: 10.1038/s41598-025-29880-9 (PMC12770318; doi:10.1038/s41598-025-29880-9)
Supplement: Supplementary file 1 — Supplementary Material 1 [file 41598_2025_29880_MOESM1_ESM.docx]

**ELECTRONIC SUPPLEMENTARY MATERIAL**

**Table S1.** Literature comparison of various heterostructure catalysts for the photocatalytic degradation of different targets.

| **S. No.** | **Photocatalyst Material** | **Pollutant** | **Illumination conditions** | **k**  **(min^-1^)** | **Degradation**  **(%)** | **Degradation**  **Time (h)** | **Reusability Cycles** | **Ref.** |
| --- | --- | --- | --- | --- | --- | --- | --- | --- |
| 1 | 5ZWO/SPI (5% ZnWO_4_ nanorods on sulfur-doped polyimide) | Methyl Orange (MO)  Methylene Blue (MB) | Simulated sunlight via XL-300 W xenon lamp | 0.00368  0.0231 | 78.54  96.04 | 6  2 | Five | ^S1^ |
| 2. | ZnWO_4_/Bi_5_O_7_I | Tetracycline (TC) | Xenon lamp simulating sunlight (visible-light irradiation) | 0.05351 | 96.8 | 1 | Ten | ^S2^ |
| 3. | ZnWO_4_/MIL-53(Fe) heterojunction composite | Tetracycline (TC) | 300 W xenon lamp with UVIRCUT420 filter (emission: 420-700 nm) | 0.01458 | 96.2 | 0.06 | Five | ^S3^ |
| 4. | 5% Cu@Cu_3x_P (hollow core-shell Cu@Cu_3x_P nanostructure with Cu vacancies) | 4-Nitrophenol (4NP)  Rhodamine B (RhB) | Xenon lamp with a 420 nm cut-off filter (simulated sunlight) | 1.38  - | 99.8  85.5 | 2  2.5 | Five | ^S4^ |
| 5. | 25% ZnWO_4_/NiWO_4_ nanocomposite | Tetracycline hydrochloride (TC-HCl) | 200 W iodine tungsten lamp (simulated visible light) | - | 90.7 | 2 | Four | ^S5^ |
| 6. | ZnCeS-ZnWO_4_ heterostructure nanocomposite | Methylene Blue (MB)  Rhodamine B (RhB)  Safranin-O (SO) dyes | Direct sunlight | 0.0693  0.0588  0.0528 | 99.7  99.1  99.0 | 1.1  1.1  1.1 | Eight | ^S6^ |
| 7. | ZnWO_4_/Ag_2_O@Ag hybrid heterostructure | Methylene Blue (MB) dye | UV light (254 nm, 6 W) | 0.05753 | 93 | 0.045 | Ten | ^S7^ |
| 8. | PdO-decorated ZnWO_4_ nanocomposite | Nitrobenzene (NB) | 500-W xenon lamp with UV cut-off filter (visible light irradiation) | 0.101 | 100 | 0.05 | Five | ^S8^ |
| 9. | 0.9 wt% PtO/ZnWO_4_ hierarchical nanospheres | Mercuric ions (Hg^2+^) | 300 W Xenon lamp (visible light, <400 nm blocked) | 0.064 | 100 | 0.05 | Five | ^S9^ |
| 10 | BiOBr/ZnWO_4_ heterojunction | Rhodamine B (RhB) dye | Visible light irradiation | 0.135 | 98.3 | 0.025 | - | ^S10^ |
| 11 | ZnWO_4_/CoWO_4_/g-C_3_N_4_ ternary nanocomposite | Methylene Blue (MB) dye | 500W Xenon lamp (Visible light irradiation) | 0.0191 | 94.6 | 2 | Four | ^S11^ |
| 12 | 9 wt% CuO/ZnWO_4_ | Hexavalent Chromium | Visible light irradiation | 0.1620 | 100 | 0.03 | Five | ^S12^ |
| 13 | Ov-ZnWO_4_/Ag-In_2_S_3_ heterojunction | Doxycycline hydrochloride (DCL.HCl) | 500 W halogen lamp | 0.0334 | 94.91 | 1.5 | Four | ^S13^ |
| 14 | ZnWO_4_/PFAC (PFAC:Plantain Flower Activated Carbon) | Rose Bengal (RB)  Rhodamine B (RhB) | Visible light (500 W tungsten lamp) | - | 99.9  99.2 | 0.045  0.045 | Five | ^S14^ |
| 15 | ZnWO_4_/FeWO_4_ composite | Tetracycline hydrochloride (TC-HCl) | Simulated sunlight (iodine tungsten lamp) | 0.0166 | 86 | 2 | Four | ^S15^ |
| 16 | ZnWO_4_ nanocrystals (biosynthesized using fruit extract) | Methylene Blue (MB) dye | Visible light (Hg lamp, 160 W) | - | 98.4 | 1.5 | - | ^S16^ |
| 17 | ZnWO_4_/g-C_3_N_4_ nanocomposite | Methylene Blue (MB)  Rhodamine B (RhB) | Visible light irradiation using a 500 W Xe lamp with λ>420 nm cut-off filter | 0.021  0.018 | 92.9  88.8 | 2  2 | - | ^S17^ |
| 18 | Polymeric carbon nitride/ZnWO_4_ | Tetracycline (TC) | Simulated visible light from a 350 W Xenon lamp with λ>420 nm | 0.014 | 95.4 | 2.6 | Four | ^S18^ |
| 19 | Cu_3_P/TiN | Sulfamethoxazole (SMX) | Visible light (1000 W xenon lamp) | 0.0102 | 90 | 2.5 | Five | ^S19^ |
| 20 | BiOIO_3_/ZnWO_4_ | Tetracycline (TC) | Simulated sunlight (300 W xenon lamp) | 0.0185 | 87.85 | 1.4 | Five | ^S20^ |
| 21 | Ag_2_MoO_4_/ZnWO_4_ | Methylene Blue (MB) | Simulated sunlight (300 W xenon lamp) | 0.10199 | 99.8 | 1 | Four | ^S21^ |
| 22 | ZnWO_4_/TiO_2_/MoS_2_ | Methylene Blue (MB) dye | 500 W high-pressure xenon lamp (UV irradiation) | 0.0296 | 99 | 2 | Three | ^S22^ |
| 23 | ZnWO_4_/BiPO_4_ | Rhodamine B (RhB)  Methyl Blue (MB)  Basic Fuchsin (BF) | 5 W LED lamp (spectral range: 410-780 nm) | 0.02368  0.00844  0.01836 | 91.4  92.8  96.5 | 1.4  6  3 | Three | ^S23^ |
| 24 | ZCS/Cu_3_P/AgCl composite (ZCS:Zinc-doped Chitosan Biochar) | Rhodamine B (Rh B) | 300 W Xenon lamp with λ>420 nm (visible light) | 0.346 | 98.76 | 0.02 | Five | ^S24^ |
| 25 | ZnWO_4_/F–TiO_2_ composite | Methyl orange | Metal halide lamp (280-780 nm) | - | 93.5% | 0.03 | Three | ^S25^ |

**S1. Chemicals and Instruments:**

Chemicals such as sodium hypophosphite (NaH_2_PO_2_), copper nitrate trihydrate (Cu (NO_3_)_2_·3H_2_O), Sodium hydroxide (NaOH), Zinc Chloride (ZnCl_2_), Sodium tungstate (Na_2_WO_4_), ethanol, Ethylene Glycol Di methacrylate (EGDMA), Azobisisobutyronitrile (AIBN), Dry DMF, were procured from sigma Aldrich and used without any advanced purification. For the pH adjustments during the photocatalysis process, buffers of 0.2 M, chloroacetic acid-acetic acid (pH 1-3), sodium acetate-acetic acid (pH 4-6), ammonium acetate-ammonia (pH 7-8), and ammonium chloride–ammonia (pH 9-10) were utilized. The model pollutant, the fluoroquinolone-based moxifloxacin (MOX), was purchased from Sigma-Aldrich. Ultrapure deionized water was utilized for all synthesis and treatment processes.

The X-ray diffraction (XRD) patterns were carried out on a BRUKER D8 Advance model powder X-ray diffractometer instrument using monochromatic Cu*Kα* radiation (k = 1.5418 Å) to analyze the crystallinity, and the subsequent diffraction peaks were cross-indexed with the standard International Centre for Diffraction Data (ICDD) database. A standard liquid-based UV-Vis spectrophotometer is insufficient for analyzing solid samples; therefore, an indirect measurement was performed using diffuse reflectance spectroscopy (DRS) to determine the absorption properties of the solid samples (JASCO V760 UV-Vis spectrophotometer). Its main feature is based on the efficient reflection of light by a non-absorptive background, usually a white BaSO_4_ coating. By this technique, the energy band gap of the synthesized nanocomposites was determined. This configuration enabled accurate measurement of the nanocomposite's light-absorption characteristics. Fourier transform infrared (FT-IR) spectra were recorded using a Thermo Scientific (Nicolet iS10) FT-IR spectrophotometer to determine chemical bonding and identify functional groups in the prepared nanocomposites. The synthesized photocatalysts' electron-hole pair recombination and charge-carrier lifetimes were measured using a Jasco FP-8550 photoluminescence spectrofluorometer (PLS). Electrochemical impedance spectroscopy (EIS) was used to analyze the charge transfer effects of surface modification, conducted in a 0.1 M [K_3_Fe(CN)_6_] and KCl electrolyte using a CH1920C electrochemical workstation. The nanocomposite surface morphology and its distribution throughout the polymer monolith were visualized using field-emission scanning electron microscopy (FE-SEM). FE-SEM images enabled precise characterization of the nanocomposites' surface properties and the resulting monolithic photocatalyst. Chemical elemental mapping with detailed element distribution was performed using an energy-dispersive X-ray spectrometer on a FEI Quanta FEQ 200 instrument. A high-resolution transmission electron microscope (HR-TEM) coupled with selected-area electron diffraction (SAED) was used at 200 kV on a Tecnai G2 20 S-Twin instrument to collect structural information on the synthesized photocatalysts. The N_2_ adsorption and desorption isotherm analysis was performed using a Quanta Chrome ASiQwin model pore-size analyzer. The specific surface area and pore size characteristics of the sample were calculated using Brunauer- Emmett-Teller (BET) and Barret–Joyner–Halenda (BJH) theoretical models, respectively. The chemical oxidation state and surface elemental composition of the CZ-20 NC and CZ-20@PEM photocatalysts were characterized using XPS analysis on a Kratos Analytical Axis 165 model. The Horiba Scientific Nanopartica SZ-100 model was employed for the zeta potential measurements. The Heber Scientific HIPR-MP400 annular photoreactor, equipped with a tungsten-filament lamp, was deployed to investigate the photocatalytic degradation of MOX drug molecules. The high-resolution mass spectrometer (HRMS, Waters, Xevo G2-XS QToF) was deployed to identify the photoproducts of the photocatalytically degraded MOX drug using the CZ-20@PEM photocatalyst. To ensure data reliability and reproducibility, we have performed multiple replicates for each experiment throughout the manuscript.

**S2**. **Optimization of Cu_3_P- ZnWO_4_ Nanocomposites:**


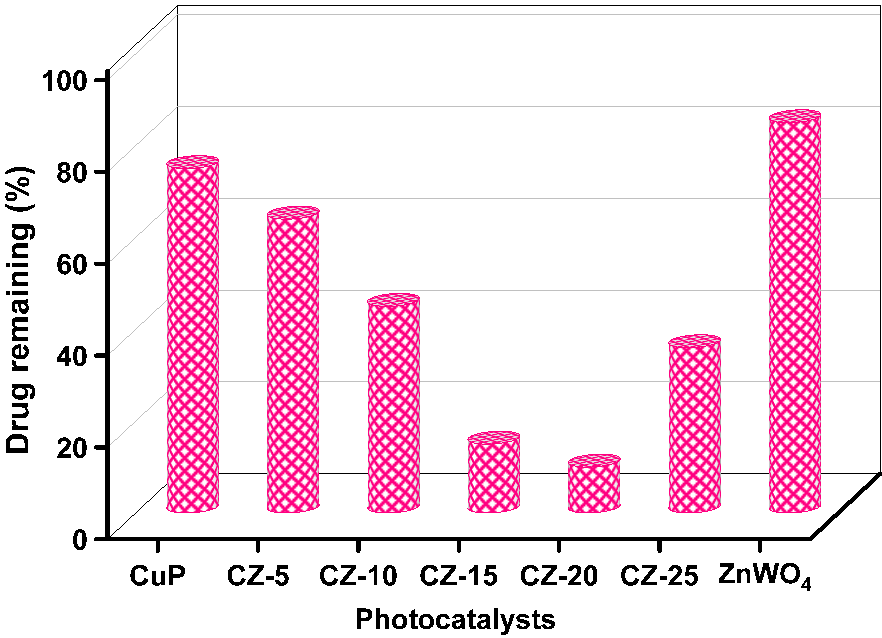


**Fig S1.** Optimizing the stoichiometry ratio of CZ NCs for maximizing photocatalytic efficiency.

During photocatalysis studies, the MOX samples and specific amounts of CZ NCs were equilibrated in the dark for 0.5 h before visible-light irradiation. In the absence of Cu_3_P inclusion, the degradation efficiency of pristine ZnWO_4_ was only 15%, as it cannot efficiently capture visible light, with ultraviolet light constituting only a tiny fraction. For pristine Cu_3_P, the efficiency was only 25% due to its narrow energy band gap, leading to rapid recombination of e^-^/h^+^ pairs despite its visible-light absorption. However, a heterojunction of CZ NC enhanced the photocatalytic efficiency, with CZ-20 NC exhibiting maximum photocatalytic degradation for MOX, as shown in **Fig. S1**. For CZ NCs, the visible-light-induced photocatalytic performance followed the order of CZ-20 (90%) > CZ-15 (79%) > CZ-25 (64%) > CZ-10 (55%) > CZ-5 (36%) > Cu_3_P (25%) > ZnWO_4_ (15%), with the parenthesis values indicating their degradation efficiency for MOX molecules. It was observed that the MOX degradation profile varied among different ratios of CZ NCs, which confirmed the significance of CZ NC stoichiometry during heterostructure formation. The superior photocatalytic activity of CZ-20 NC has been attributed to its ability to extend light absorption across the visible spectrum, facilitating the efficient separation of photoexcited charge carriers. The heterojunction formation resulted in intermediate energy levels that decreased the rate of e^-^/h^+^ recombination, thereby enhancing the visible-light-induced photocatalytic performance among all the synthesized CZ NCs.

**S3. Adsorption-Desorption and Isotherm Studies:**

The adsorption-desorption equilibrium of MOX was attained within 60 minutes under dark conditions for the Cu_3_P, ZnWO_4_, CZ-20 NC, and CZ-20 PEM, as illustrated in **Fig. S2(a)**. Among these materials, CZ-20@PEM exhibited the highest adsorption capacity, achieving 3.2% MOX removal in the absence of light. This superior adsorption behavior, combined with a photodegradation efficiency exceeding ≥99.4% under visible-light irradiation, is attributed to the synergistic interaction between CZ-20 and PEM. The synergetic effect enhances the surface area and promotes the formation of a continuous mesoporous framework. These results indicate that, although adsorption plays a relatively minor role compared to photocatalysis, the enhanced adsorption ability of CZ-20@PEM contributes to the overall MOX removal efficiency.

Additionally, adsorption isotherms **(Fig. S2(b))** were employed to describe the relationship between the quantity of MOX adsorbed per unit mass of photocatalyst and its equilibrium concentration in solution. To evaluate adsorption behavior, both the Langmuir and the Freundlich models were applied. The experimental data showed an excellent correlation with the Freundlich isotherm (R^2^ = 0.98), significantly outperforming the Langmuir model. This strong agreement highlights the heterogeneous surface characteristics of the photocatalyst. The equation expresses the Freundlich isotherm,

$q_{e}=K_{F}C_{e}^{\frac{1}{n}}$

Where *q_e_* (mg/g) is the equilibrium adsorption capacity, *C_e_* (mg/L) is the equilibrium concentration of the adsorbate. *K_F_* (mg/g). (L/mg)^1/n^ is the Freundlich constant indicating adsorption capacity, and *1/n* is the heterogeneity factor representing adsorption intensity. The equation gives the linearized form,

$logq_{e}=\log K_{F}+\frac{1}{n}logC_{e}$

From the linear regression analysis of the Freundlich plot, the slope and intercept were obtained as 1.365 and 1.28, respectively. From the linear regression of the Freundlich plot, the slope and intercept were determined to be 1.365 and 1.28, respectively. This value corresponds to *n* = 0.732 and *K_F_* = 19.05 (mg/g)(L/mg)^1.365^. Since *1/n* > 1, the adsorption process is unfavourable at higher MOX concentrations, reflecting a decline in adsorption efficiency with increasing solute levels due to saturation of adsorption sites. Nevertheless, the relatively high *K_F_* value demonstrates a strong adsorption affinity at lower MOX concentrations. This indicates that adsorption is most effective at low concentrations, further corroborating the heterogeneous nature of the photocatalyst surface and suggesting the predominance of weak physisorption interactions.

**
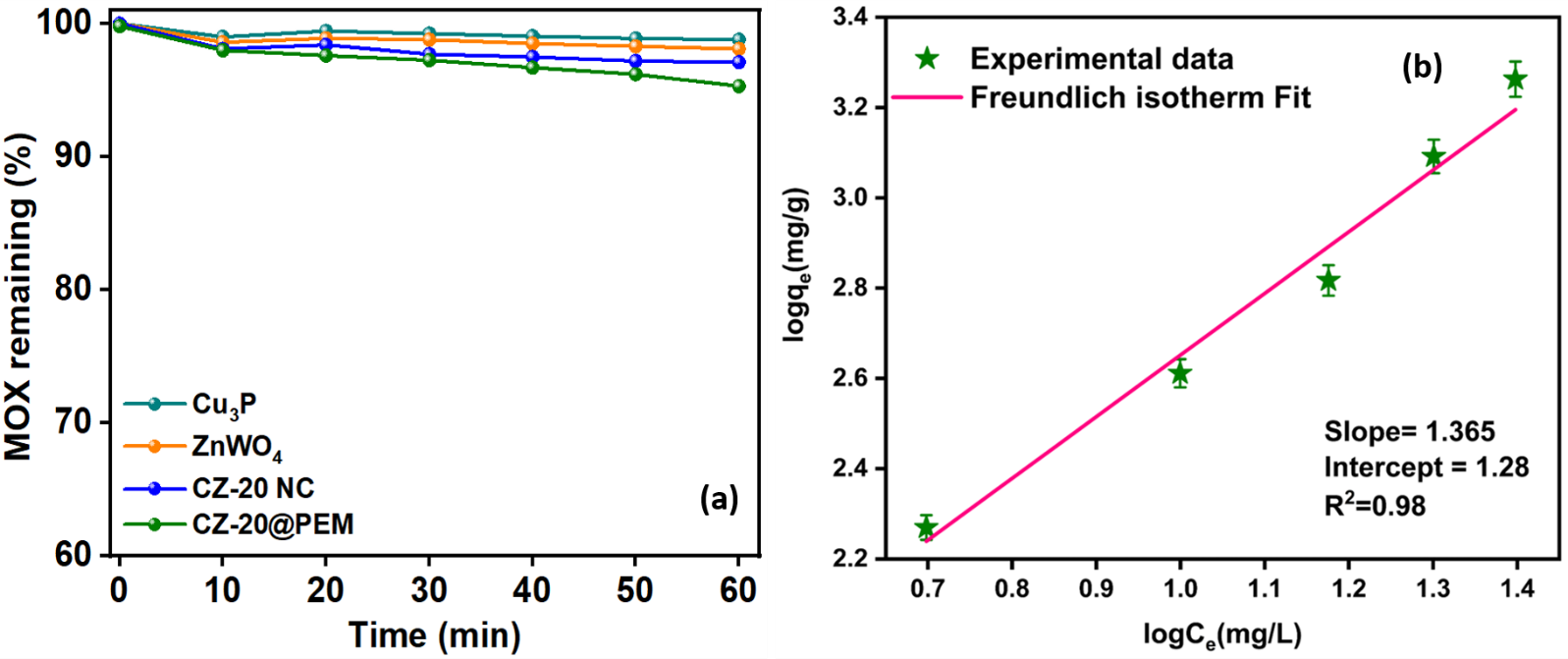
**

**Fig S2. (a)** Adsorption curve of MOX molecules on Cu_3_P, ZnWO_4_, CZ-20 NC, and CZ-20 PEM photocatalyst in dark conditions and **(b)** Freundlich linear fit plot for the CZ-20@PEM photocatalyst-MOX drug system.

**S4**. **Schematic Representation of the Synthesis of CZ NCs and CZ-20@PEM Photocatalyst:**

**
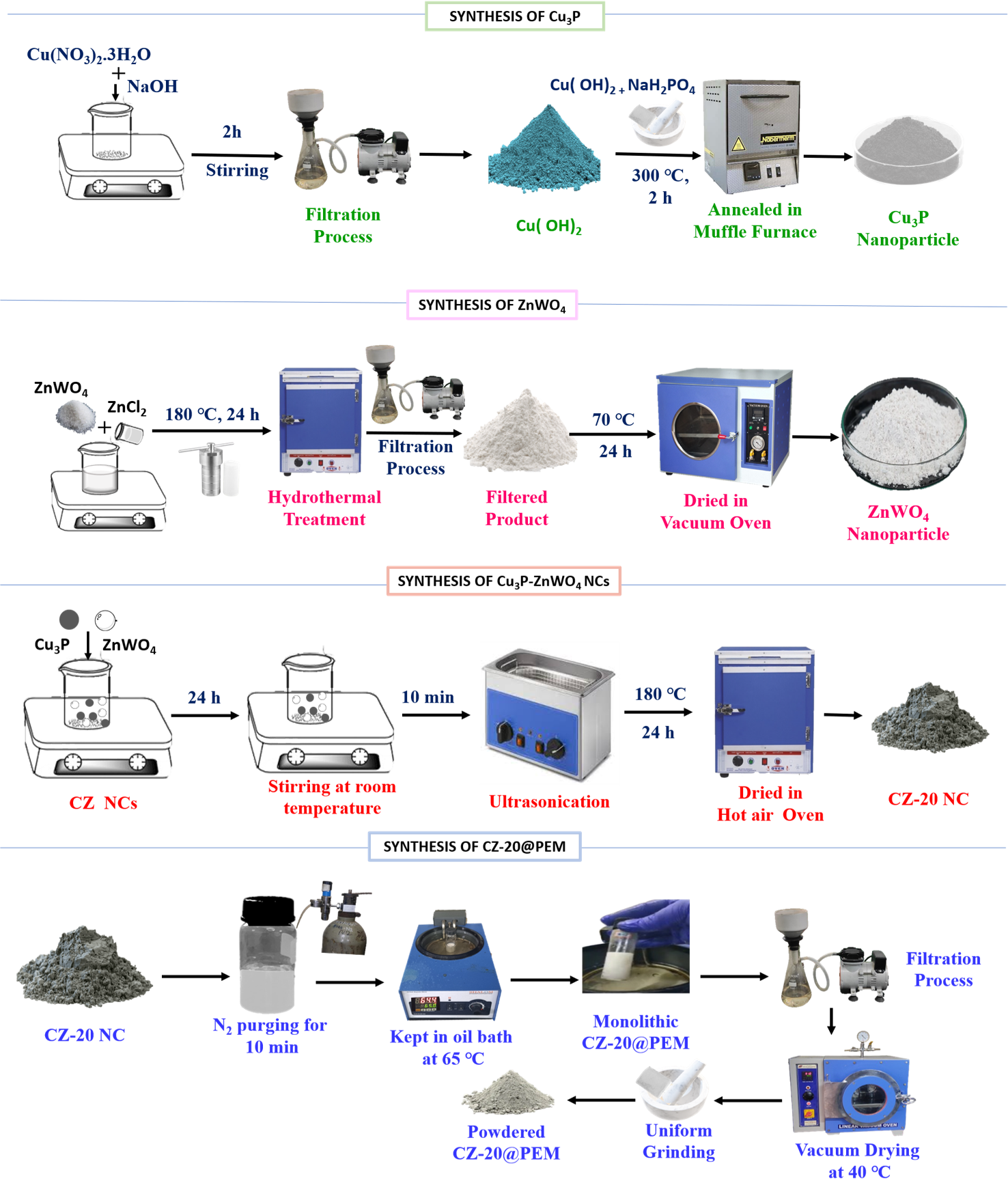
**

**Scheme S1.** Schematic description of the synthesis of Cu_3_P, ZnWO_4_ nanoparticle, CZ NCs, and CZ-20@PEM photocatalyst.

**S5. FE-SEM Analysis:**

**
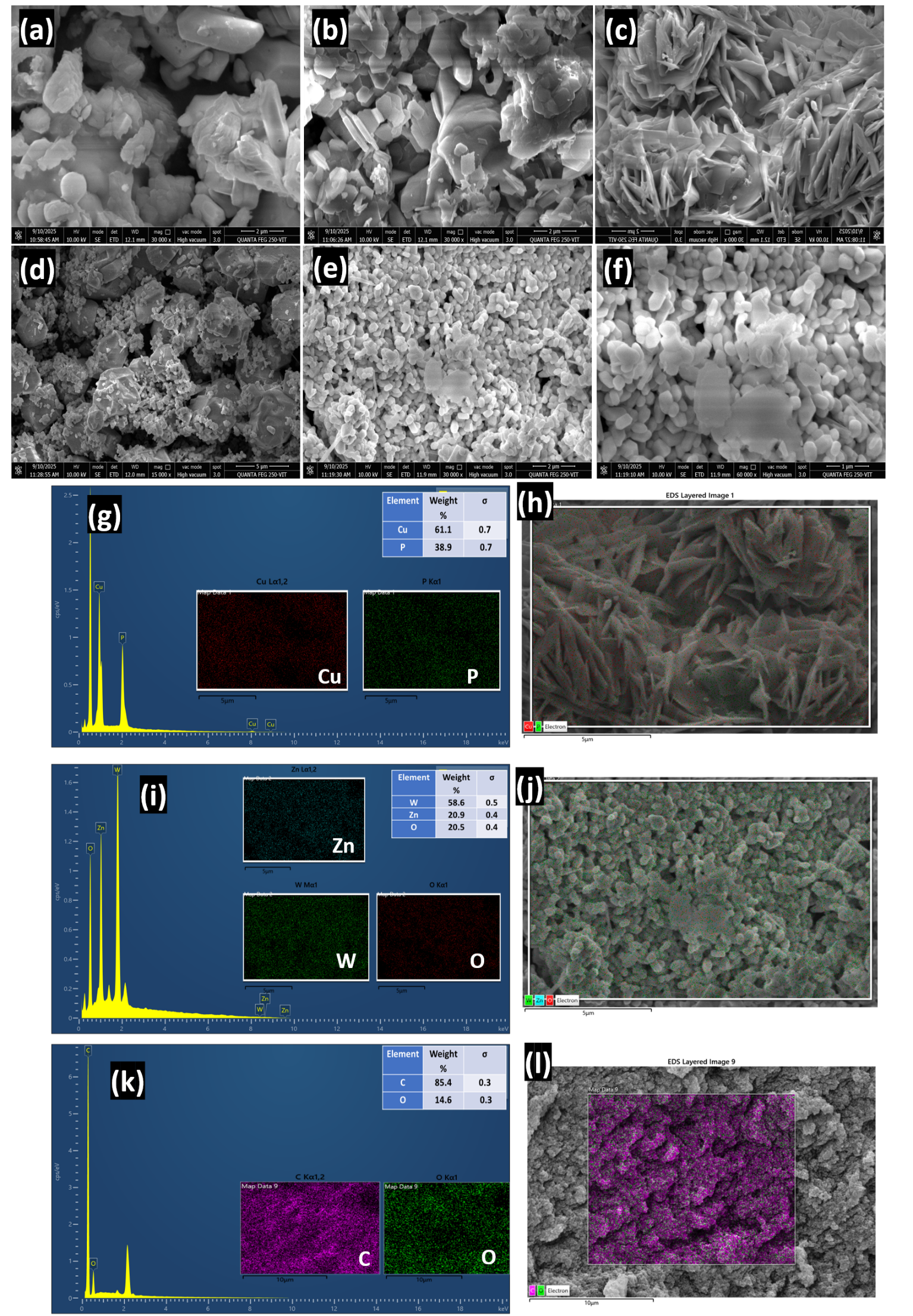
**

**Fig S3.** FE-SEM images of **(a-c)** Cu_3_P and **(d-f)** ZnWO_4_ nanoparticles_,_ **(g)** EDX spectrum of Cu_3_P with corresponding elemental mapping **(h)** (inset), **(i)** EDX spectrum of ZnWO_4_ with corresponding elemental mapping **(j)** (inset), and **(k)** EDX spectrum of PEM with corresponding elemental mapping **(l)** (inset).

The FE-SEM, EDX, and elemental mapping analyses of Cu_3_P and ZnWO_4_ are presented in **Fig. S3**. The FE-SEM images **(Fig. S3 (a-c))** reveal the formation of small Cu_3_P hexagonally plate-like nanoparticles with flower-like clusters, while **Fig. S3 (d-f)** displays ZnWO_4_ nanoparticles with a tiny spherical uniform morphology. The distinct morphologies of the two materials may favour close interfacial contact and synergistic charge transport when combined into a CZ-20 nano heterostructure. The EDX spectrum of Cu_3_P **(Fig. S3 (g)** confirms the presence of Cu and P in the expected stoichiometric ratio, further supported by elemental mapping **(Fig. S3 (h), inset),** which illustrates the uniform distribution of Cu and P. Similarly, the EDX spectrum of ZnWO_4_ **(Fig. S3 (i))** validates the presence of Zn, W, and O in appropriate proportions, with the corresponding elemental mapping **(Fig. S3 (j), inset)** confirming their homogeneous spatial distribution within the ZnWO_4_ nanostructures.

**S6. HR-TEM Analysis:**

**
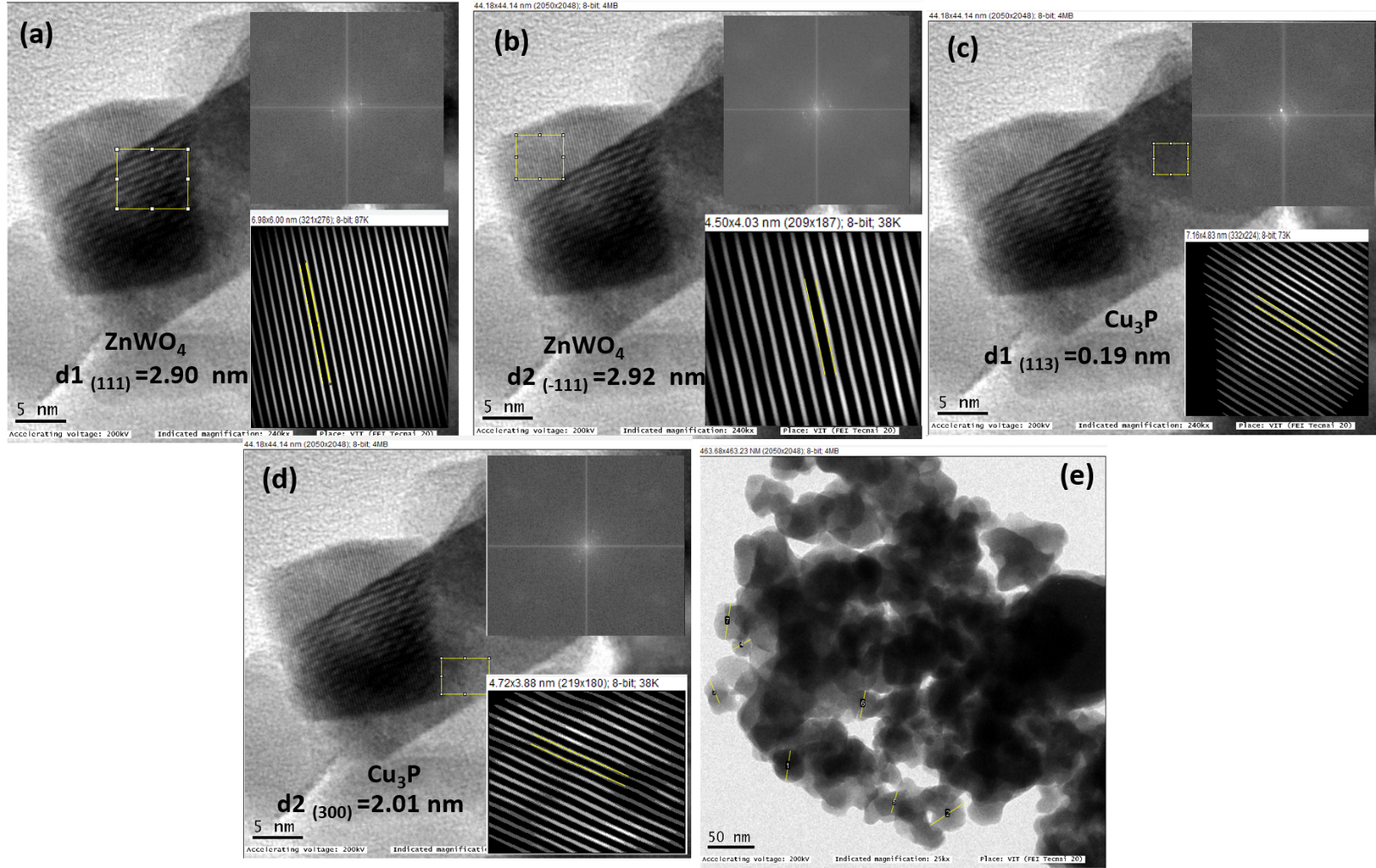
**

**Fig S4.** **(a-d)** HR-TEM analysis of CZ-20 NC insets showing FFT and inverse FFT, and **(e)** HR-TEM analysis of CZ-20 NC (Grain size measured).

**Table S2:** Measured interplanar spacings and angles of the CZ-20 NC.

| **Spot** | **d-Spacing (nm)** | **Rec. Pos.(1/nm)** | **Degrees to Spot** | **Degrees to the x-axis** | **Amplitude** |
| --- | --- | --- | --- | --- | --- |
| 1 | 0.2955 | 3.503 | 0 | -142.69 | 14342.28 |
| 2 | 0.1929 | 5.183 | 65.6 | 77.79 | 5628.71 |
| 3 | 0.2926 | 3.406 | 136.02 | -167.17 | 14408.11 |
| 4 | 0.2027 | 4.884 | 103.28 | 114.03 | 4562.33 |
| 5 | 0.1006 | 9.94 | 66.74 | 76.66 | 1153.5 |
| 6 | 0.09552 | 10.47 | 46.67 | 96.72 | 1206.87 |
| 7 | 0.08903 | 11.23 | 22.39 | 121.01 | 529.21 |

The HR-TEM images with FFT and inverse FFT analyses are shown in **Fig. S4**, where the FFT patterns display sharp diffraction spots corresponding to the observed lattice fringes, facilitating accurate measurement of interplanar spacings and angles, as well as assignment of the relevant (hkl) indices. The analysis confirmed the crystalline nature of Cu_3_P and ZnWO_4,_ and ensured the reliable identification of lattice planes in the CZ-20 NC. The inverse FFT further isolated individual planes, thereby reducing projection-related uncertainties. Although the HRTEM images in **Fig. S4(a-d)** predominantly exhibit single sets of fringes, the reciprocal-space information derived from FFT provides precise verification of plane orientation and spacing. The measured interplanar distances (2.90, 2.92, 0.19, and 2.01 nm) are consistent with the (1 1 1), (−1 1 1), (1 1 3), and (3 0 0) planes of ZnWO_4_ and Cu_3_P, respectively, and are in good agreement with the ICDD reference values as presented in p-XRD. The indexed lattice fringes have been labeled in the micrographs with their corresponding Miller indices. These images clearly reveal lattice fringes along two distinct crystallographic directions, and the combined real-space (HR-TEM) and reciprocal-space (FFT) analyses provide strong confirmation of the phase assignment. The corresponding d-spacings were measured and used to validate the crystalline phases of both Cu_3_P and ZnWO_4_ in the CZ-20 NC. The grain size distribution has been depicted in **Fig. S4(e)**, and the corresponding values are summarized in **Table S1.**

**S7. TrPL Analysis:**

Time-resolved photoluminescence spectroscopy (TrPL) has been used to investigate the dynamics of charge-carrier properties in CZ NCs and in the CZ-20@PEM photocatalyst. This technique enables the study of fluorescence decay, allowing us to understand the lifetime of charge carriers generated by photoexcitation. The TrPL analysis revealed that the photocatalyst's shorter average lifetime indicates more efficient electron-hole pair separation and reduced recombination, consistent with the increased photocatalytic activity observed for the CZ-20@PEM photocatalyst, followed by CZ-20 NC, compared to other CZ NCs and pristine ZnWO_4_. The results of the TrPL analysis confirm that enhanced charge-carrier dynamics are crucial for the photocatalyst's performance under visible-light irradiation. Notably, data from photoluminescence (PL) and diffuse reflectance spectroscopy (DRS) were consistent with the observed photocatalytic activity of the CZ-20 NC-loaded PEM photocatalyst, which showed a significantly reduced PL intensity. A weakened PL response indicates that electron-hole recombination has been suppressed. For charge carriers generated by light to be effective in photocatalysis, they require a long enough lifespan and the ability to move to the catalyst surface, where they can react with water and oxygen molecules to produce reactive oxygen species (ROS). Therefore, it is essential to understand both the spatial distribution and temporal evolution of these charge carriers. Time-resolved photoluminescence spectroscopy provides significant insights into carrier dynamics by recording recombination events between mobile electrons in the conduction band or shallow traps and immobile hole polarons in deep traps, as well as the transport of electrons from the core of the heterostructure nanocomposite to the surface or depletion region. The decay profiles of CZ NCs and CZ-20 dispersed in the PEM matrix have been depicted in **Fig. S5,** with average lifetimes of 744 and 950 ns for CZ-20 NC and CZ-20@PEM, respectively. The prolonged lifetimes of electrons and holes in CZ-20@PEM were attributed to the efficient suppression of charge-carrier recombination, enabling more photogenerated electrons and holes to participate in surface reactions. The PEM framework's high surface-to-volume ratio supports this improvement by providing greater accessibility to active sites, facilitating more efficient charge separation and transfer, and thereby enhancing overall photocatalytic performance.


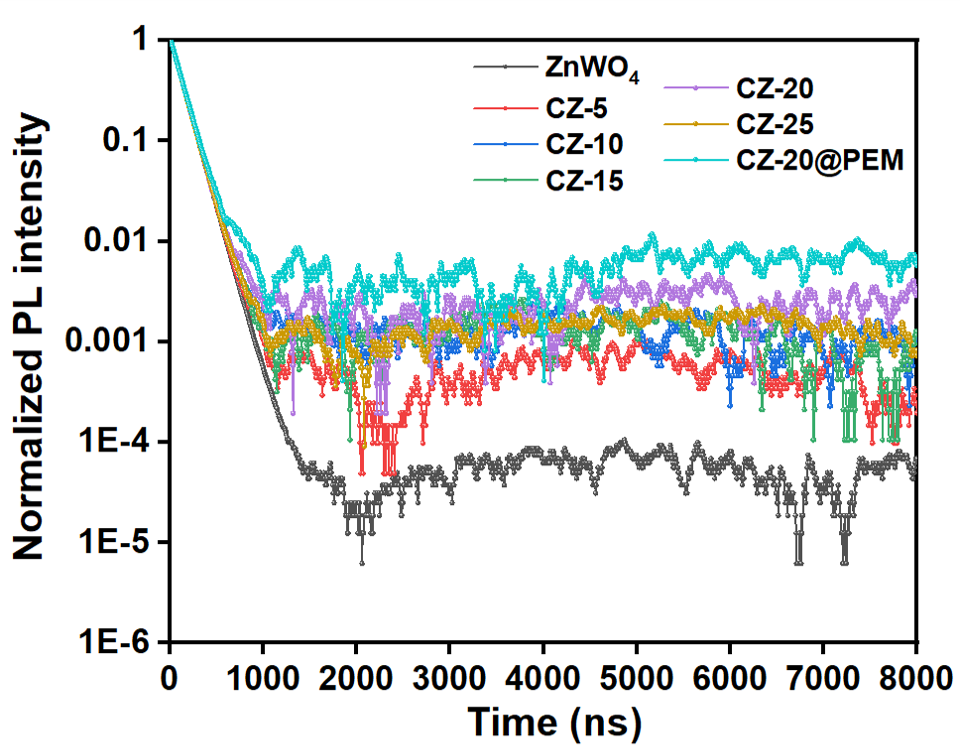


**Fig S5.** TrPL analysis of ZnWO_4_, CZ-NCs and CZ-20 PEM

**S8. EIS Analysis:**

Electrochemical analyses were employed to elucidate further the mechanisms underlying the enhanced photocatalytic activity by examining the dynamics of electron-hole separation. Impedance spectroscopy measurements were performed using electrochemical techniques to investigate the charge transfer characteristics of the synthesized photocatalysts. **Fig. S6** presents Nyquist plots for pristine ZnWO_4_, CZ nanocomposites (NCs), and the CZ-20@PEM photocatalyst. From the results obtained, the semicircular arc located in the high-frequency area signifies the charge transfer resistance at the interface between the electrode and electrolyte. At the same time, the linear segment in the low-frequency area mirrors capacitive properties. Of all the materials examined, ZnWO_4_ exhibited the greatest arc radius, which is a sign of significant resistance to interfacial charge transfer. Its behaviour was attributed to its wide bandgap and inherently low electrical conductivity, which impedes effective charge separation and carrier mobility.

In contrast, the CZ-20 nanocomposite demonstrated a smaller arc radius than pristine ZnWO_4_, suggesting that the creation of a heterojunction substantially enhances charge carrier transport, decreases recombination rates, and improves interfacial contact. The CZ-20@PEM composite exhibited the smallest arc radius among all other samples, indicating the lowest interfacial charge transfer resistance and the most efficient charge separation. The performance enhancement of this material can be attributed to its porous heterostructure, enlarged surface area, and exceptional electron mobility, which collectively enable the efficient separation and migration of photogenerated charge carriers. The EIS spectrum of CZ-20@PEM also showed a significantly smaller semicircular arc than that of pure ZnWO_4_, indicating enhanced interfacial conductivity. In combination, EIS and photoluminescence spectroscopy results suggest that incorporating CZ-20 NCs into the PEM matrix greatly facilitates the separation of photogenerated charge carriers and hinders recombination, which are critical factors contributing to the superior photocatalytic efficiency of the CZ-20@PEM system.


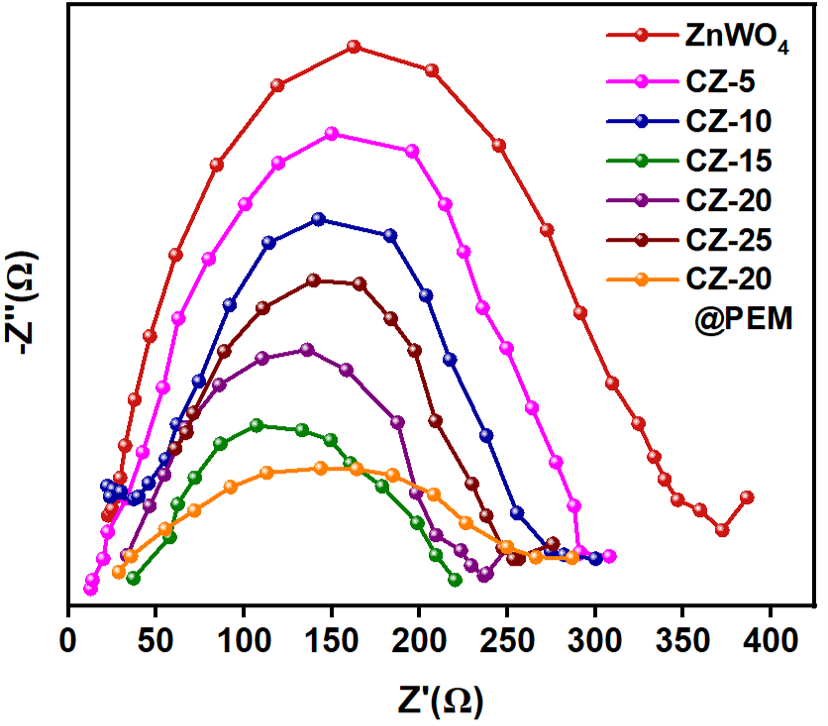


**Fig S6.** EIS analysis of ZnWO_4_, CZ-NCs and CZ-20 PEM

**S9. XPS Analysis – Comparison Spectra:**


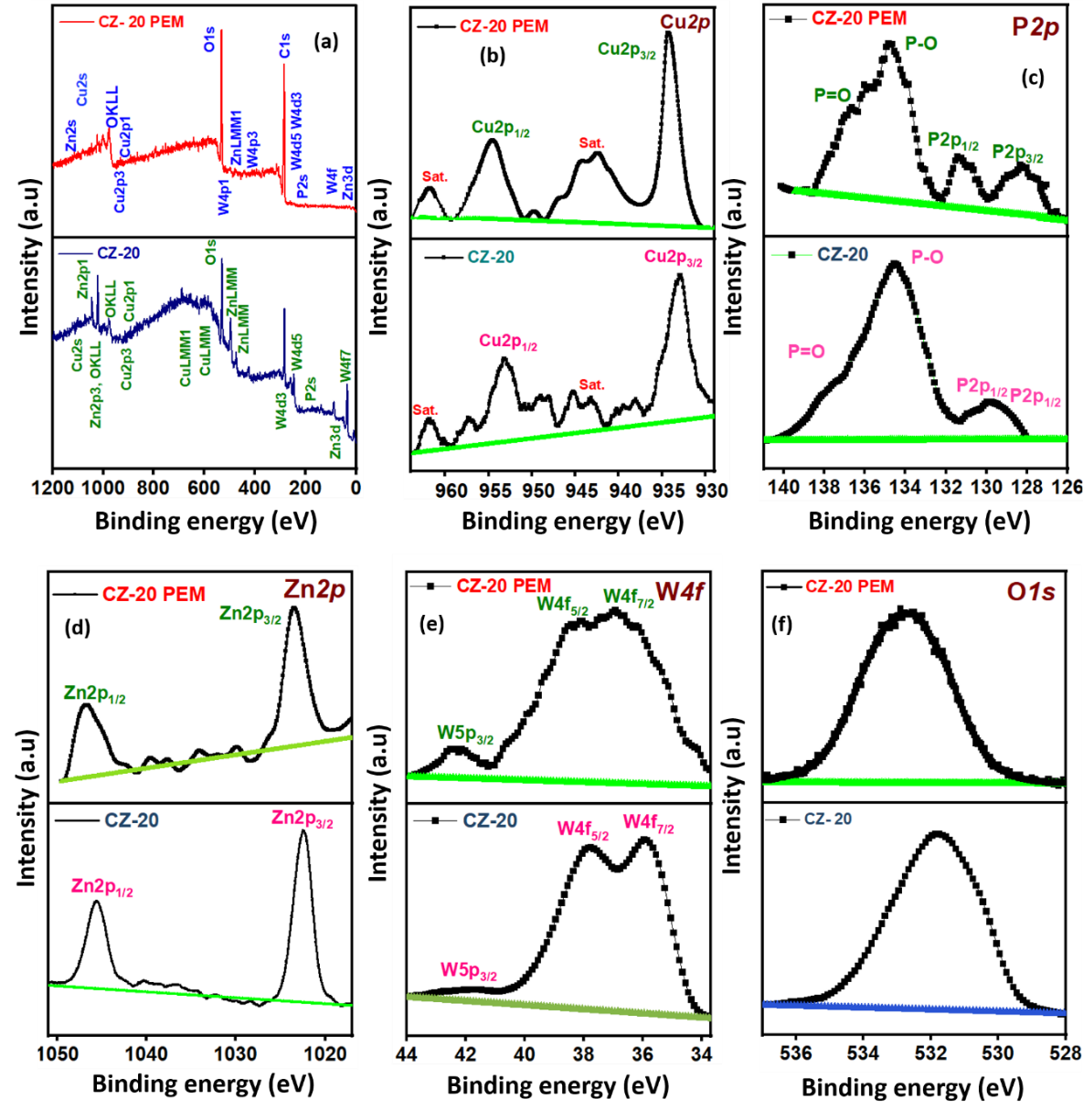


**Fig S7.** XPS analysis of CZ-20 and CZ-20 PEM – A comparative study.

**S10**. **p-XRD, BET & BJH and FE-SEM Analysis of Reused Photocatalyst:**


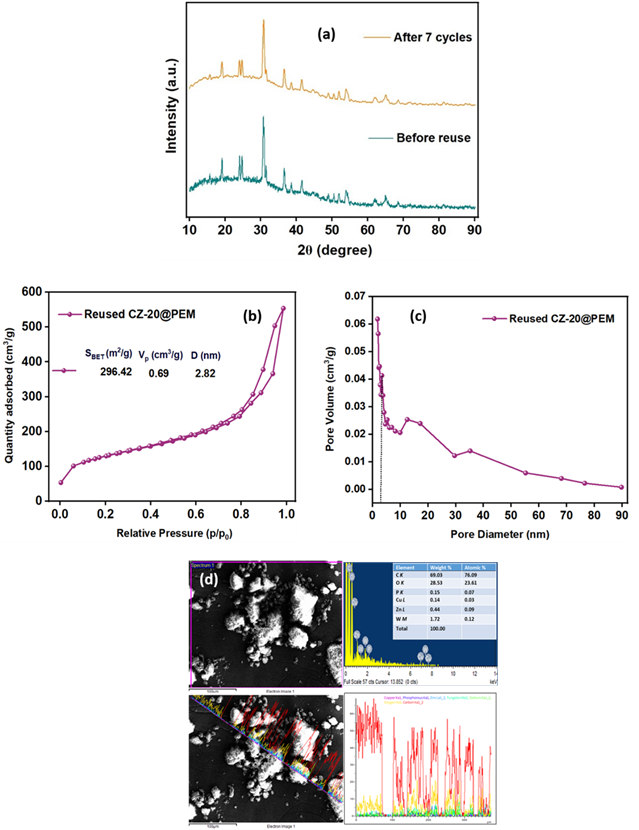


**Fig S8. (a)** p-XRD, **(b)** BET, **(c)** BJH, and **(d)** FE-SEM-EDX & elemental line profile analysis of the reused CZ-20@PEM photocatalyst.

**S11. Real-Time Application Studies using CZ-20@PEM Photocatalyst:**

The photocatalytic performance of the CZ-20@PEM catalyst for the degradation of MOX was further evaluated using not only simulated lab water but also environmental water and industrial wastewater samples. Before initiating the photocatalytic experiments, each water sample was intentionally spiked with a known concentration of MOX drug molecules., the degradation efficiency of CZ-20@PEM was assessed in various water matrices, including simulated lab water (SW), lake water (LW) collected from VIT Vellore (Tamil Nadu, India), tap water (TW) collected from VIT Vellore (Tamil Nadu, India), and industrial wastewater (IW) collected from Ranipet Sipcot (Tamil Nadu, India). As illustrated in **Fig. S9,** simulated lab water demonstrated the highest degradation efficiency, reaching 99.4%. In contrast, the efficiencies observed in lake water, tap water, and industrial water were 82.7%, 89.2%, and 78.5%, respectively. The diminished efficiency of these natural and industrial samples can be attributed to the presence of diverse ionic species and complex, diverse organic and inorganic constituents that impede photocatalytic activity. Interfering substances may contend for active sites on the catalyst's surface, remove reactive oxygen species, or prevent light from penetrating, all of which decrease the overall degradation efficiency. Nonetheless, the CZ-20@PEM catalyst still achieved a significant degradation performance even in these complex aqueous environments, highlighting its practical utility for real-world wastewater treatment. The robustness of the CZ-20@PEM system stems from its enhanced surface area, strong interfacial charge separation, and stable photo response. The porous structure of this material also enables better mass transfer of pollutants, and the combined effect of the CZ-20 nanocomposite active sites and the PEM support lengthens the lifetime of photogenerated carriers. The catalyst demonstrated good photocatalytic efficiency, suggesting it can be applied to various types of water. Overall, the obtained results demonstrate the potential of CZ-20@PEM as a promising candidate for large-scale, environmentally friendly photocatalytic applications.


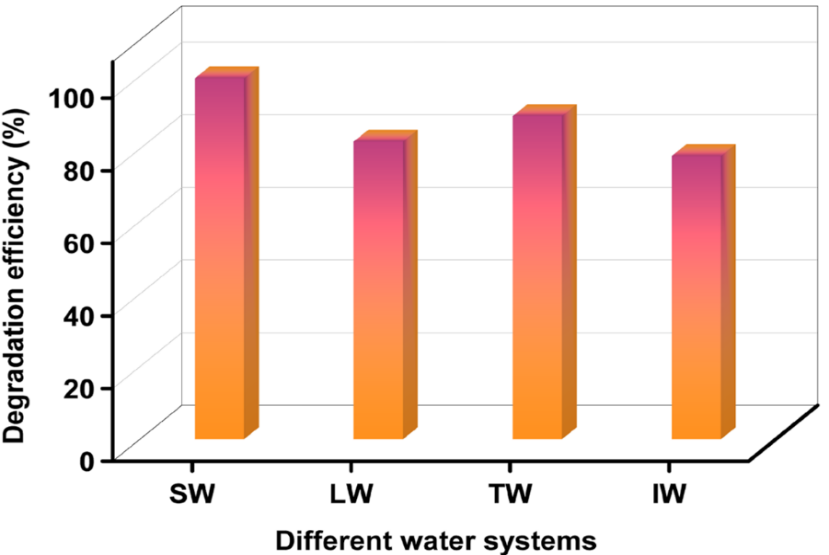


**Fig S9.** Photocatalytic efficiency of the CZ-20 PEM toward MOX degradation in different water systems.

**S12. HR-MS Analysis of Photo-catalytically Degraded MOX Drug:**

The HR-MS analysis of the pristine MOX is depicted in **Fig. S10.** Subsequently, the HR-MS analysis was employed to identify the photoproducts of MOX derived from the visible light-induced photocatalysis of CZ-20@PEM, and the possible degradation pathway is also depicted in **S11(a-c)**. A summarized list of photoproducts from MOX degradation is presented in **Scheme S2**.

**
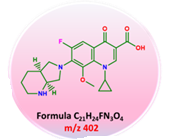

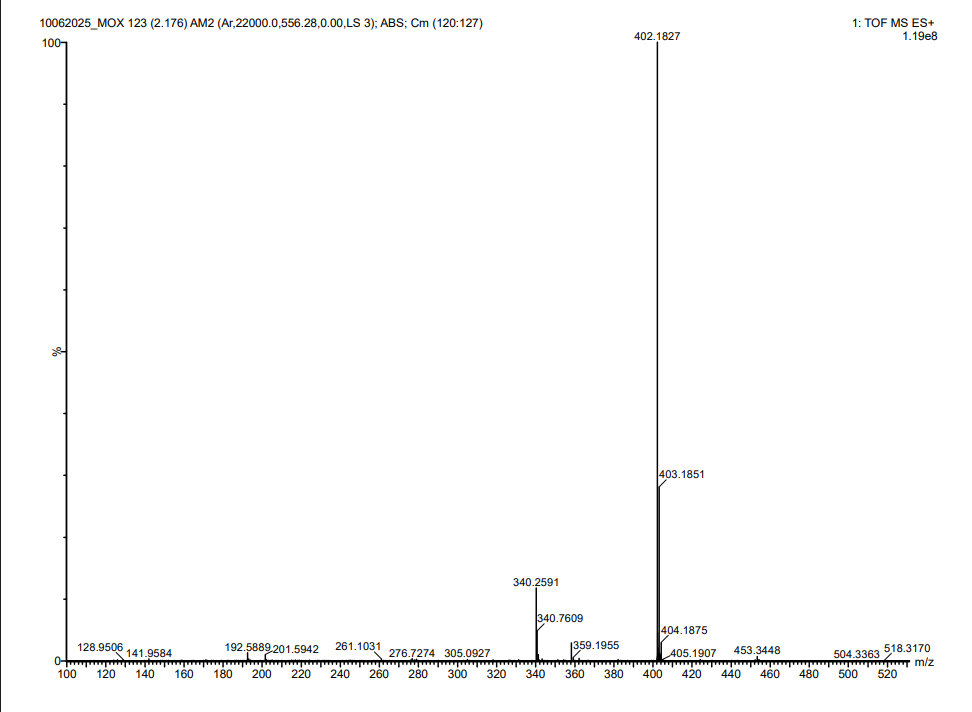
**

**Fig S10.** HR-MS analysis of non-photocatalyzed/pristine MOX drug compound.


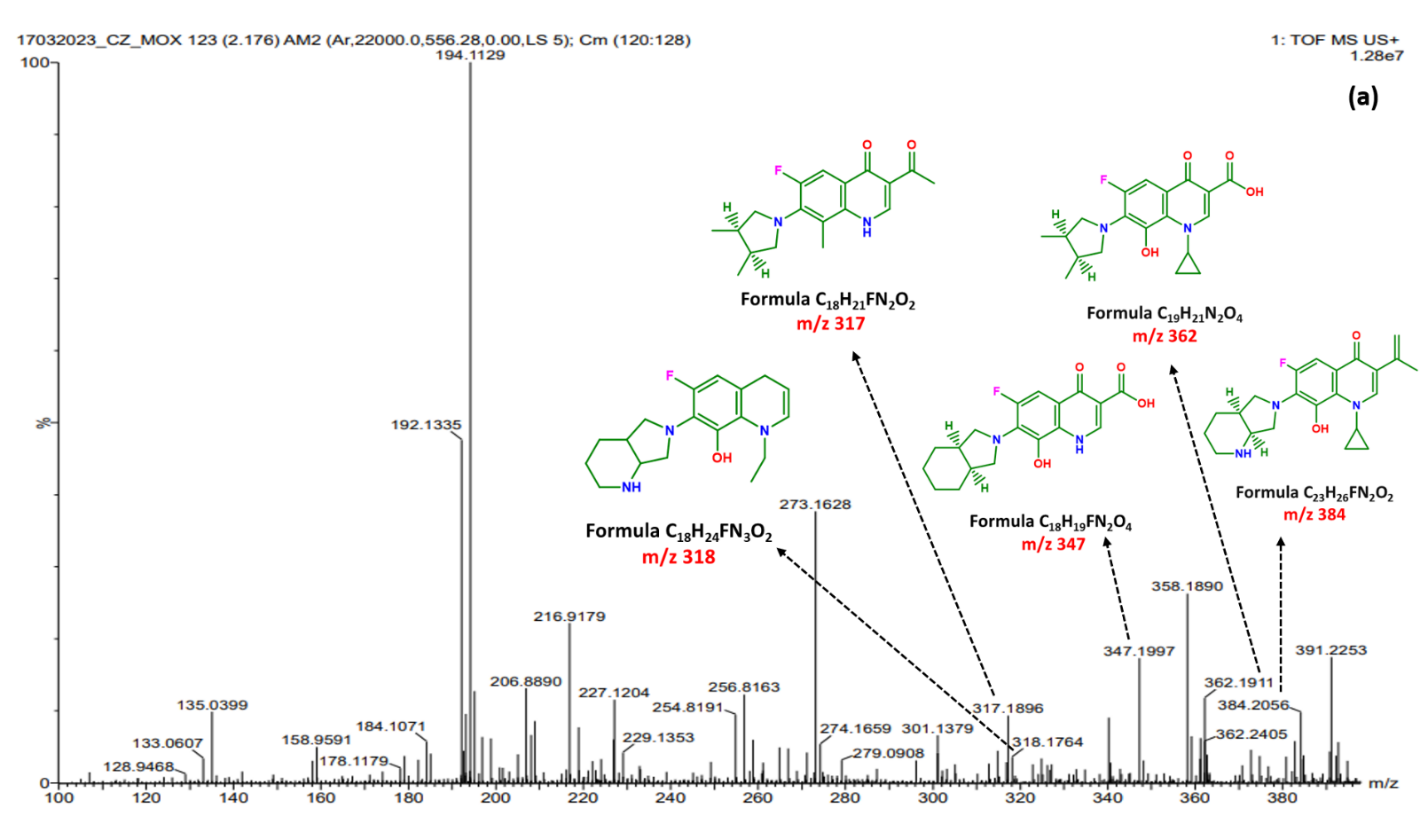


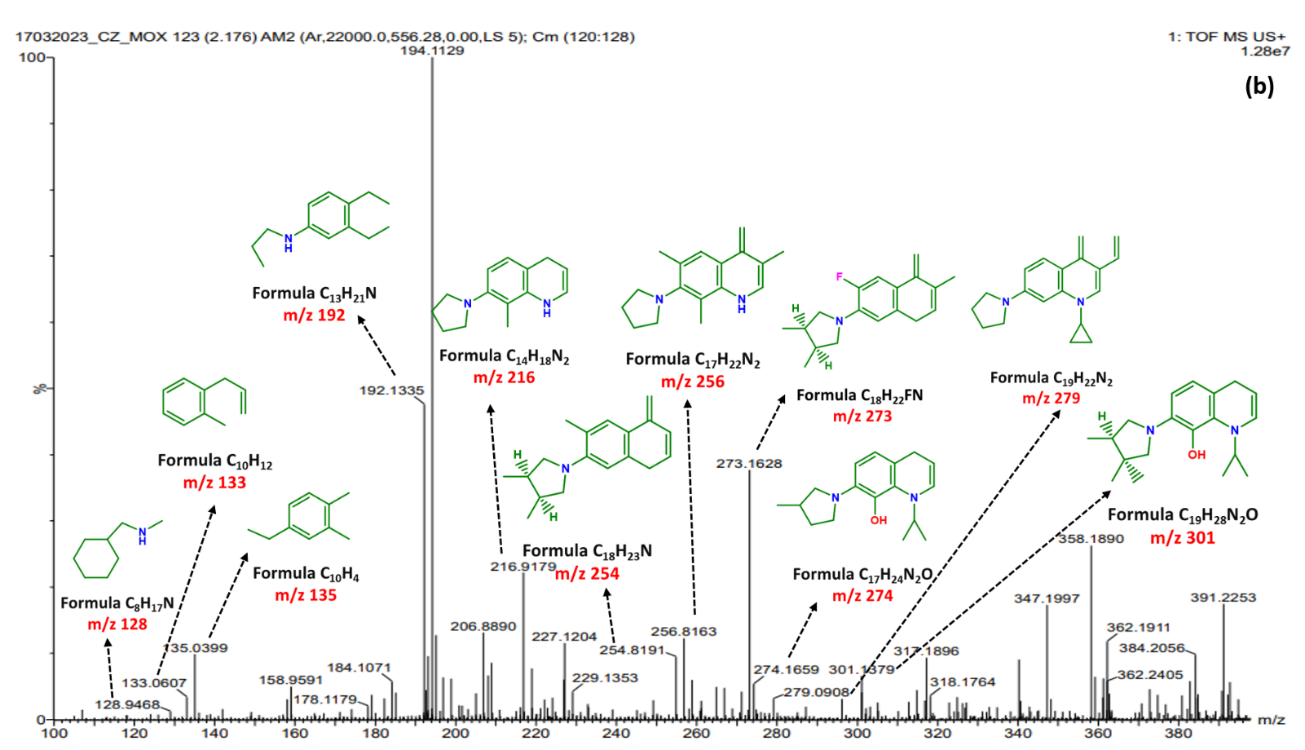


**Fig S11. (a&b)** HRMS data of photoproducts from the photocatalytic degradation of MOX drug molecules using CZ-20 PEM photocatalyst under visible light irradiation.


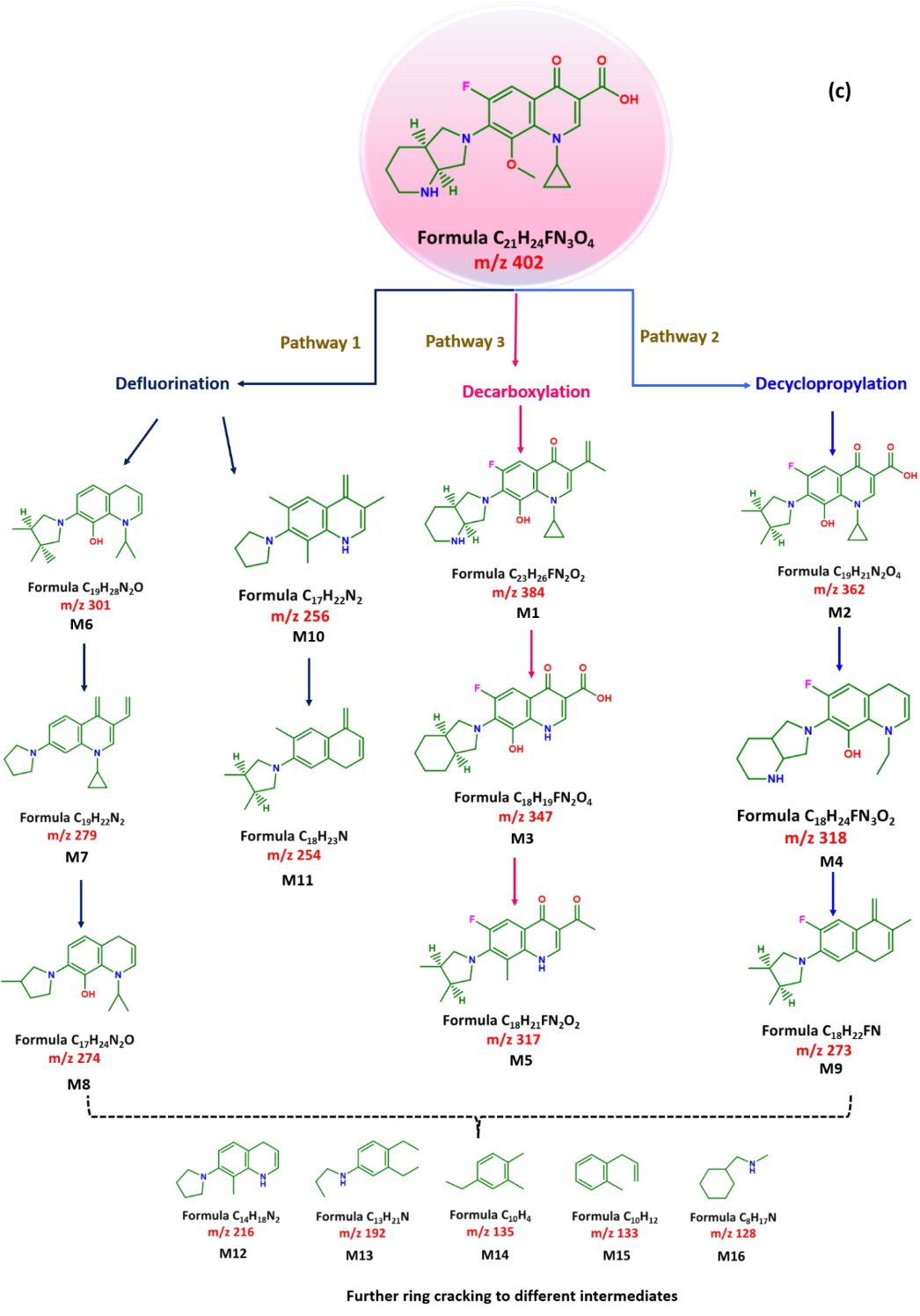


**Fig S11. (c)** Proposed pathway for the photocatalytic degradation of MOX drug molecules using CZ-20 PEM photocatalyst under visible light irradiation.

**
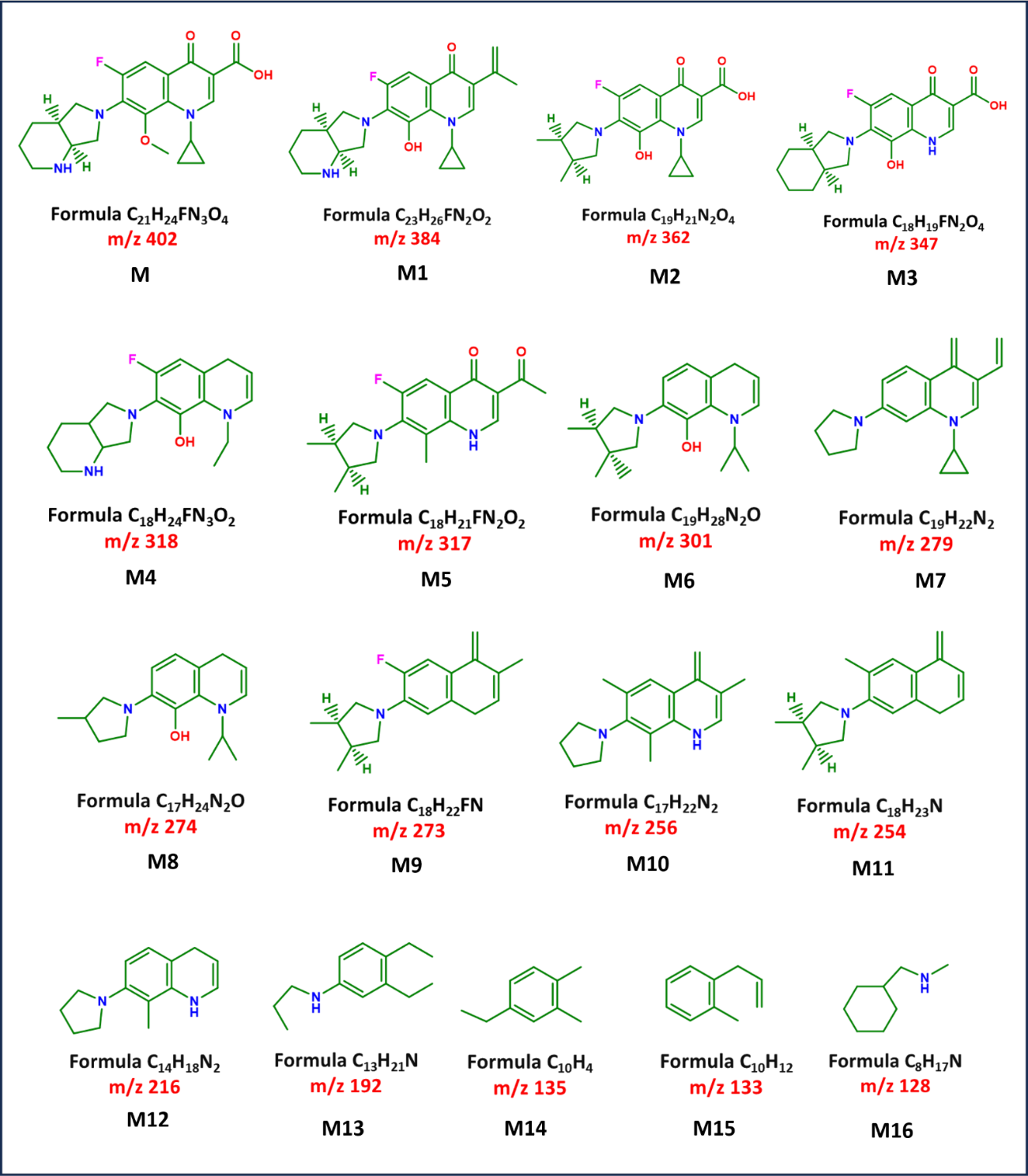
**

**Scheme S2.** Photoproducts of MOX drug from the visible-light-induced photocatalytic degradation using CZ-20@PEM photocatalyst.

**References**

S1. Jia, X., Liu, B., Chen, Y. & Wang, X. Nanoconstructing ZnWO_4_ sulfur-doped polyimide Z-scheme heterojunction with improved charges transfer for enhanced solar photocatalysis. *Sol. Energy* **301,** 113981 (2025).

S2. Zhao, M., Zhang, S., Shi, L. & Lei, C. Efficient photocatalytic degradation of tetracycline by Z-scheme ZnWO_4_ /Bi_5_O_7_ I heterojunction. *Mater. Sci. Semicond. Process.* **185,** 108935 (2025).

S3. Jiang, Z., Yang, J., Chen, F., Song, Y. & Tang, Y. Construction of a novel ZnWO_4_ / MIL-53 ( Fe ) heterojunction photocatalyst for the boosted tetracycline degradation under visible-light irradiation. *Mater. Sci. Semicond. Process.* **200,** 109982 (2025).

S4. Kou, Y., Wang, K., Wumaer, M., Guo, C., Tian, B., Zhang, L., Akram, N. & Wang, J. Synthesis of hollow Cu @ Cu_3−X_P core-shell nanostructure as dual-functional catalyst with copper vacancy for enhancing chemical reduction and photocatalytic performance. *Appl. Surf. Sci.* **589,** 153031 (2022).

S5. Wang, X., Lin, S., Cui, N., Qi, K., Liu, S. & Khan, I. Synthesis of ZnWO_4_/NiWO_4_ photocatalysts and their application in tetracycline hydrochloride degradation and antibacterial activities. *J. Taiwan Inst. Chem. Eng.* **157,** 105408 (2024).

S6. Munawar, T., Fatima, S., Mujasam, K., Shahid, M., Mukhtar, F., Hussain, S., Alim, S., Koc, M. & Iqbal, F. Enhanced charge carriers separation/ transportation via S-scheme ZnCeS – ZnWO heterostructure nanocomposite for photodegradation of synthetic dyes under sunlight. *Mater. Chem. Phys.* **314,** 128938 (2024).

S7. Geetha, G. V., Vadivel, G., Das, G. M., Saha, S., Kuppusamy, M. & Krishnasamy, S. Synergistic enhancement of plasmon induced photocatalytic and photoelectrochemical performance on ZnWO_4_/Ag_2_O @ Ag. *Mater. Chem. Phys.* **328,** 129959 (2024).

S8. Al-namshah, K. S. & Mohamed, R. M. PdO-decorated ZnWO_4_ heterojunctions : One-pot approach and quick solar-light-driven photocatalytic reduction of nitrobenzene with excellent recyclability. *Ceram. Int.* **51,** 3820–3829 (2025).

S9. Ayad, N., Ashour, A., Baoum, A. A., Shawky, A. & Mohamed, R. M. Promoted visible-light driven photoreduction of mercuric ions over hydrothermally synthesized platinum oxide-supported zinc tungstate hierarchical nanospheres. *Mater. Sci. Eng. B* **323,** 118800 (2026).

S10. Andrade, A. O. C., Henrique, L., Júnior, M. M. L., Sharma, K., Maia, M. E. H., Alves, O. C., Santos, E. C. S., Santos, C. C., Menezes, A. S. De, San-miguel, M. A., Moura, F., Longo, E. & Almeida, M. A. P. Enhanced photocatalytic activity of BiOBr/ZnWO_4_ heterojunction : A combined experimental and DFT-based theoretical approach. *Opt. Mater. (Amst).* **138,** 113701 (2023).

S11. Parasuraman, B., Shanmugam, P. & Riswana, N. Advanced engineering of smart nanomaterials : ZnWO_4_/CoWO_4_/g-C_3_N_4_ heterojunction photocatalysts for environmental and biomedical application. *J. Alloys Compd.* **1010,** 178200 (2025).

S12. Basaleh, A. S., Abdel-haleem, F. M., Mohamed, R. M. & Khedr, T. M. Novel mesoporous CuO-adorned ZnWO_4_ heterojunction for visible-light-driven elimination of hexavalent chromium. *Colloids Surfaces A Physicochem. Eng. Asp.* **722,** 137305 (2025).

S13. Soni, V., Singh, P., Ahamad, T., Katin, K. P., Kaya, S., Kumar, N., Nguyen, V., Mustansar, C. & Raizada, P. Constructing oxygen vacancy enriched ZnWO_4_/Ag-doped In_2_S_3_ visible light active S-scheme heterojunction with improved charge migration for doxycycline hydrochloride degradation in wastewater. *Colloids Surfaces A Physicochem. Eng. Asp.* **727,** 138466 (2025).

S14. Karthikadevi, S., Mullainathan, S., Begum, N. J., Manimaran, S. & Muruganantham, N. Multiple synergistic benefits of the composite partners ZnWO_4_ and activated carbon : Augmented photocatalytic performance. *Inorg. Chem. Commun.* **181,** 115204 (2025).

S15. Song, J., Yang, Y., Cui, N., Sadiq, S., Khan, I. & Liu, S. Preparation and application of ZnWO_4_/FeWO_4_ based photocatalyst : Organic pollutant degradation and microbicide. *J. Water Process Eng.* **68,** (2024).

S16. Hkiri, K., Elsayed, H., Mohamed, A., Bongani, C., Mongwaketsi, N., Gibaud, A. & Maaza, M. Promising photocatalytic activity under visible light of ZnWO_4_ nanocrystals prepared via green synthesis approach. *Mater. Today Commun.* **35,** 106355 (2023).

S17. Riswana, N., Parasuraman, B., Wang, P., Zeng, C., Cheng, Y. & Thangavelu, P. Facile construction of ZnWO_4_/g-C_3_N_4_ heterojunction for the improved photocatalytic degradation of MB, RhB and mixed dyes. *Surfaces and Interfaces* **53,** 105039 (2024).

S18. Luo, Z., Fang, Y., Chen, J. & Wu, Y. Rational design of Z-scheme configured polymeric carbon nitride-decorated ZnWO_4_ nanofibers for enhanced visible-light-driven photodegradation of antibiotics. *Surfaces and Interfaces* **54,** 105293 (2024).

S19. Shi, H., Yao, X., Lu, S., Zuo, Y., Zheng, T. & Jia, L. Photocatalytically Active Semiconductor Cu_3_P Unites with Flocculent TiN for Efficient Removal of Sulfamethoxazole. *Catalysts,* 1–13 (2023).

S20. Wei, G., Dong, F., Yuan, Z., Zhou, D., Liu, C., Wan, H., Jin, M., Shao, M. & Wan, Y. Synthesis of Type II Heterojunction BiOIO_3_/ZnWO_4_ Nanocomposites for Photocatalytic Degradation of Tetracycline. *Chemistry Select* ***10*,** 03895 (2025).

S21. Zhang, J., Ma, J., Sun, X., Yi, Z., Xian, T., Wu, X., Liu, G., Wang, X.& Yang, H. Construction of Z-scheme Ag_2_MoO_4_/ZnWO_4_ heterojunctions for photocatalytically removing pollutants. *Langmuir* ***39*,** 1159-1172 (2023).

S22. Zhang, H., Liu, X., Li, Z., Wang, F., Zhang, J., Gao, F., Zhang, P. & Wei, Z. Preparation and performance study of ZnWO_4_/TiO_2_/MoS_2_ ternary composite photocatalyst. *Journal of Materials Science* ***59*,** 38-54 (2024).

S23. Yi, Z., Yan, G., Fu, Z., Wen, M. & Hong, M. Enhanced performance for dyes degradation over ­ ZnWO_4_/BiPO_4_ heterojunction. *Ionics* 2727–2738 (2025).

S24. Li, X., Wu, S., Wang, J., Ding, Y. & Feng, X. Construction of ZCS/Cu_3_P/AgCl composites for enhanced removal of Rhodamine B : Performance and Mechanism. *Russ. J. Inorg. Chem.* **69,** 1276–1289 (2024).

S25. Liu, M., Zhang, L., Tang, J. & Deng, S. Zinc Tungstate Coupled Fluorinated Titanium Dioxide (ZnWO_4_/F-TiO_2_) composites with enhanced photocatalytic activity. *Russ. J. Phys. Chem. A* **97,** 3238–3248 (2023).
